# Hydrogen Tunnelling as a Probe of the Involvement of Water Vibrational Dynamics in Aqueous Chemistry?

**DOI:** 10.3390/molecules25010172

**Published:** 2019-12-31

**Authors:** Ana Karković Marković, Cvijeta Jakobušić Brala, Viktor Pilepić, Stanko Uršić

**Affiliations:** Faculty of Pharmacy and Biochemistry, University of Zagreb, A. Kovačića 1, 10 000 Zagreb, Croatia; akarkovic@pharma.hr (A.K.M.); vpilepic@pharma.hr (V.P.)

**Keywords:** hydrogen tunnelling, kinetic isotope effects, water vibrational dynamics, aqueous reactivity, PCET reactions, ascorbate

## Abstract

Our study of tunnelling in proton-coupled electron transfer (PCET) oxidation of ascorbate with hexacyanoferrate(III) follows the insights obtained from ultrafast 2D IR spectroscopy and theoretical studies of the vibrational water dynamics that led to the proposal of the involvement of collective intermolecular excitonic vibrational water dynamics in aqueous chemistry. To test the proposal, the hydrogen tunnelling modulation observed in the PCET reaction studied in the presence of low concentrations of various partial hydrophobic solutes in the water reaction system has been analyzed in terms of the proposed involvement of the collective intermolecular vibrational water dynamics in activation process in the case. The strongly linear correlation between common tunnelling signatures, isotopic values of Arrhenius prefactor ratios ln *A*_H_/*A*_D_ and isotopic differences in activation enthalpies ΔΔ*H*^‡^ (H,D) observed in the process in fairly diluted water solutions containing various partial hydrophobic solutes (such as dioxane, acetonitrile, ethanol, and quaternary ammonium ions) points to the common physical origin of the phenomenon in all the cases. It is suggested that the phenomenon can be rooted in an interplay of delocalized collective intermolecular vibrational dynamics of water correlated with vibrations of the coupled transition configuration, where the donor-acceptor oscillations, the motions being to some degree along the reaction coordinate, lead to modulation of hydrogen tunnelling in the reaction.

## 1. Introduction

We report a study of kinetic isotope effects (KIE) and hydrogen tunneling in PCET reaction of ascorbate with hexacyanoferrate(III) that follows recent Tokmakoff and coworker’s studies [1,2,3] where they have suggested that traditional way of conceiving solutes in water and aqueous chemical reactivity may be revised; the notion follows the insights obtained from ultrafast 2D IR spectroscopy and theoretical studies of the vibrational water dynamics [1,2,3,4,5,6,7,8,9,10]. These studies revealed the highly collective and intermolecular excitonic nature of coupled water vibrations; there are also the observations of coherent vibrational character of coupled solute and solvent when there is strong hydrogen bonding between solute and the water molecule [1,3]. Consequently, a revision has been proposed of the common picture of the interaction between a solute and the solvating water molecules in which their interaction is treated as a perturbation in the case of aqueous solutes, especially when there is strong hydrogen bonding between the solute and water molecule [1,2,3] as water vibrations are found to be delocalized, and the vibrational eigenstates associated with the solute in water may also be delocalized, with correlations that extend over its solvation shell. Therefore, the distance scale and mechanism over which concerted bimolecular chemical reactions, such as proton transfer can occur would be determined by the extent of coherent vibrational character of coupled solute and solvent. Then, in aqueous chemical reactions, these vibrational motions would also be along with dissipation the origin of activation processes.

**Scheme 1:**
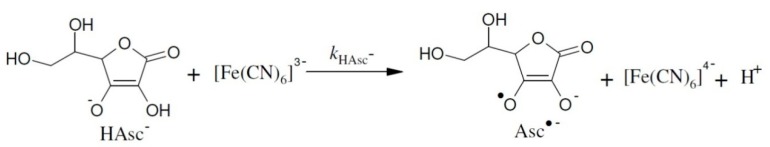
Oxidation of ascorbate monoanion with the hexacyanoferrate(III) ion. *k*_Hasc−_ is the second-order rate constant for the rate-controlling PCET process (the first step).

To date, there is no experimental report beyond the ultrafast 2D IR spectroscopy dealing with the far-reaching proposal; we start with the expectation that the PCET [11,12,13,14,15,16,17] oxidation of l-ascorbate with hexacyanoferrate(III) [18,19,20,21,22,23] ions (Scheme 1) may present a good model reaction to test the above proposal of the involvement of vibrational water dynamics in aqueous chemical reactivity. More specifically, this reaction system (1) presumably involves hydrogen bond between the water molecule and the solute (ascorbate anion) and (2) the reaction is concerted in character [19,20,21,22,23]. Furthermore, we start with the expectation that the system feature that can reflect coherent vibrational motions between coupled solute and solvent would be the modulation of hydrogen tunneling in the case of that concerted reaction as the reaction system involves proton donor-acceptor distance (DAD) oscillations, the dynamical feature related to hydrogen tunneling [23,24,25,26,27,28,29,30,31,32,33], in the PCET/enzyme-catalyzed reactions.

The key concept here is that fast, femtosecond to picosecond motions are involved during the reaction, modulating the DAD in the H-transfer [25,26,27,28,29,30,31]. As the consequence of relevant fluctuations in DAD (distance sampling of the donor-acceptor coordinate), sampling of tunnelling-ready configurations can lead to (effective) narrower barrier width thus enhancing the probability of and facilitating the tunnelling in the process [25,26,31,32,33]. In addition, the theory of the PCET reactions [16,17,24,34,35] involves the frequency of DAD oscillations in the reaction as one of the crucial dynamical parameters. Therefore, expectation is that changes in the dynamical features of the system with regard to DAD oscillations could be reflected through the modulation of the common signatures of hydrogen tunneling, the values of isotopic Arrhenius prefactor ratios *A*_H_/*A*_D_ (where H stands for reaction of ascorbate monoanion HAsc^−^ with hexacyanoferrate(III) ion in H_2_O and D for reaction of deuterated ascorbate monoanion DAsc^−^ with hexacyanoferrate(III) ion in D_2_O) and the isotopic differences in activation enthalpies ΔΔ*H*^‡^ that are well beyond its semiclassical limits [36,37,38,39,40].

We report here a study of hydrogen tunneling phenomena in the above PCET reaction (Scheme 1) performed in fairly diluted water solutions containing low concentrations of various (inert) partially hydrophobic solutes. We expect that these very diluted water solutions probably can be considered as a good representation of water, as the excitonic dynamics is critical in neat water. Furtherly, expectation is that due to collective and intermolecular character of water vibrations coupled also with the solute vibrations [1,3], the presence of low concentrations of hydrogen-bonded partially hydrophobic substrates would lead to changes in water dynamics and consequently to changes of the dynamical features of the coupled water-transition reaction configuration system with regard to DAD oscillations; then these changes could be reflected through the modulation of the common signatures of hydrogen tunneling.

Indeed, we have observed the modulation of hydrogen tunneling in our reaction in the presence of fairly low concentrations of partially hydrophobic substrates such as dioxane, acetonitrile, ethanol, and quaternary ammonium ions added into water reaction solution. The evidence we report here point strongly to a causal connection between the collective intermolecular vibrational dynamics of water and the dynamics of coupled transition reaction configuration that is reflected by the hydrogen tunneling modulation in the above mentioned PCET interaction of ascorbate monoanion with hexacyanoferrate(III) ions.

## 2. Results and Discussion

In this study we have examined the temperature dependence of KIE and the modulation of hydrogen tunnelling induced by the partially hydrophobic solutes in the PCET [11,12,13,14,15,16,17] interaction of ascorbate monoanion with hexacyanoferrate(III) ions (see Scheme 1) in order to gain information related to the assumed involvement of the collective vibrational water dynamics to aqueous chemistry. Since the collective excitonic vibrational water dynamics is critical in neat water [1,2,3,4], the reaction was performed in the fairly diluted water reaction solutions containing low concentrations of the partially hydrophobic solutes such as dioxane, acetonitrile, ethanol, and quaternary ammonium ions. We have assumed that these very diluted water solutions can be considered as an acceptable representation of neat water.

The analysis of temperature dependence of KIE is considered to be the gold standard for experimental study of the hydrogen tunneling phenomena [31,32]. Recently, we have observed the common signatures of hydrogen tunnelling i.e., the values of isotopic Arrhenius prefactor ratios *A*_H_/*A*_D_ and the isotopic differences in activation enthalpies ΔΔ*H*^‡^ that are well beyond the semiclassical limits [36,37,38,39,40] in this ascorbate concerted PCET reaction performed in water reaction solution in the presence of substantial concentrations of some partially hydrophobic solutes [20,21,22]. However, there is a question whether these solutions containing the substantial concentrations of the solutes can be compared with the neat water solutions at all, and whether and to what extent the solutes can change driving force of the reaction Δ*G*°, and the equilibrium proton donor-acceptor distance and the effective frequency Ω of donor-acceptor mode (R-mode), the parameters that, accordingly to theory [16,17,24,34,35] can influence the magnitude of KIE observed. Consequently, we have performed our experiments in the fairly diluted water solutions of the partially hydrophobic substrates in order to avoid the effects of presence of substantial concentrations of the substrates on bulk solvent properties and the vibrational excitonic dynamics of water, and KIE and hydrogen tunneling in the reaction. Moreover, as already mentioned above, the fairly diluted solutions are applied since the strong OH stretch-intermolecular mode coupling are only available in neat H_2_O where the excitonic nature of the OH stretch is critical [1,2,3,4]. Indeed, the finding that the point for neat water falls on the same straight line obtained correlating isotopic ratios of Arrhenius prefactors ln *A*_H_/*A*_D_ and the isotopic differences of Arrhenius activation energies *E*_a_^D^–*E*_a_^H^ (or isotopic differences in activation enthalpies ΔΔ*H*^‡^) for a number of various fairly diluted water solutions of the partially hydrophobic solutes suggests strongly that the above assumptions can be in the right direction.

### 2.1. The Temperature Dependence of Kinetic Isotope Effects. The Correlation of Isotopic Differences in the Activation Enthalpies ΔΔH^‡^ (D,H) and the Isotopic Differences in Entropies ΔΔS^‡^ (D,H)

**Figure 1:**
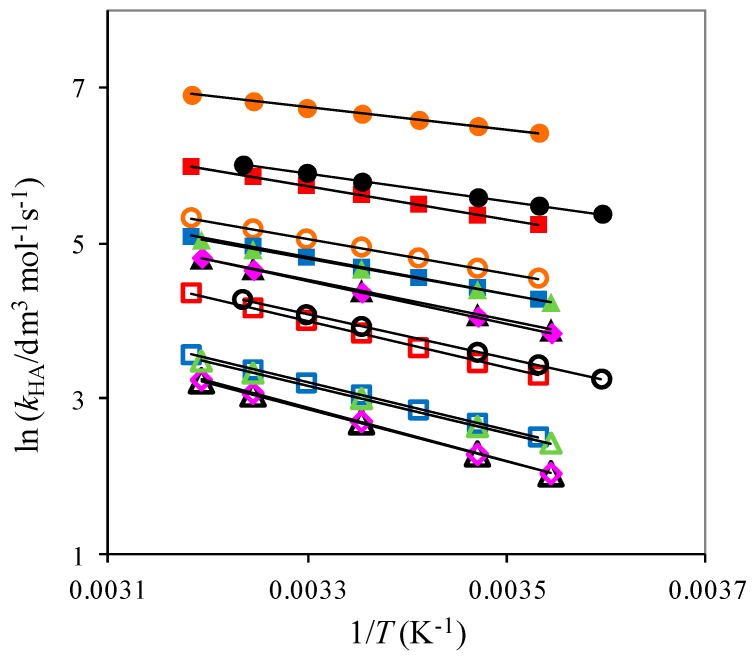
Arrhenius plot on the reaction of ascorbate and hexacyanoferrate(III) ion in presence of low concentrations of different quaternary ammonium salts in H_2_O (filled symbols) and D_2_O (hollow symbols). Key: green triangle, 0.005 M TMACl; black triangle, 0.005 M TPACl; magenta diamond, 0.005 M TBACl; blue square, 0.01 M TEACl; orange circle, 0.006 M PQCl_2_; red square, 0.1 M AChCl; black circle, 0.1 M BTMACl. Abbreviations as noted in Table 1.

**Figure 2:**
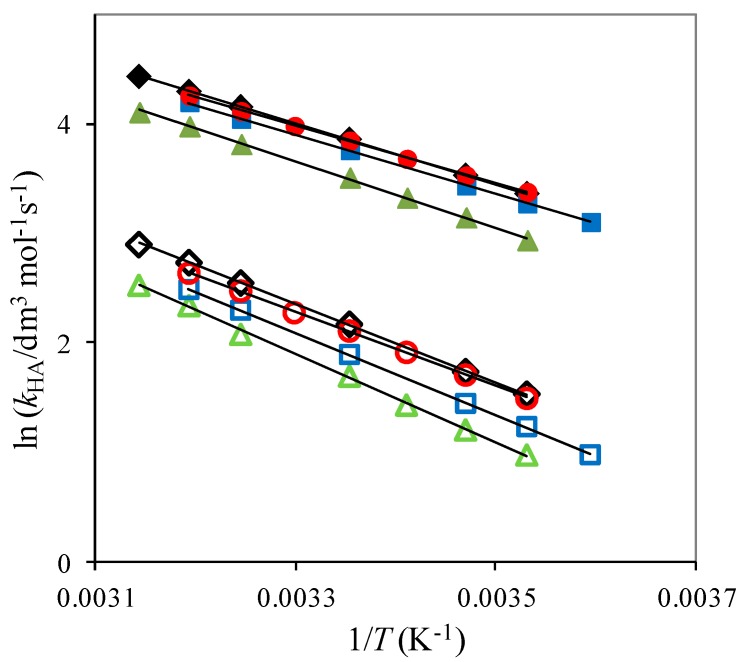
Arrhenius plot on the reaction of ascorbate and hexacyanoferrate(III) ion in different organic cosolvent—H_2_O (filled symbols) and organic cosolvent—D_2_O mixtures (hollow symbols). Key: black diamond, 1,4-diox:water = 0.05:0.95 *v*/*v*; green triangle, 1,4-diox:water = 0.10:0.90 *v*/*v*; blue square, MeCN:water = 0.10:0.90 *v*/*v*; red circle, EtOH:water = 0.10:0.90 *v*/*v*. Abbreviations as noted in Table 1.

**Figure 3:**
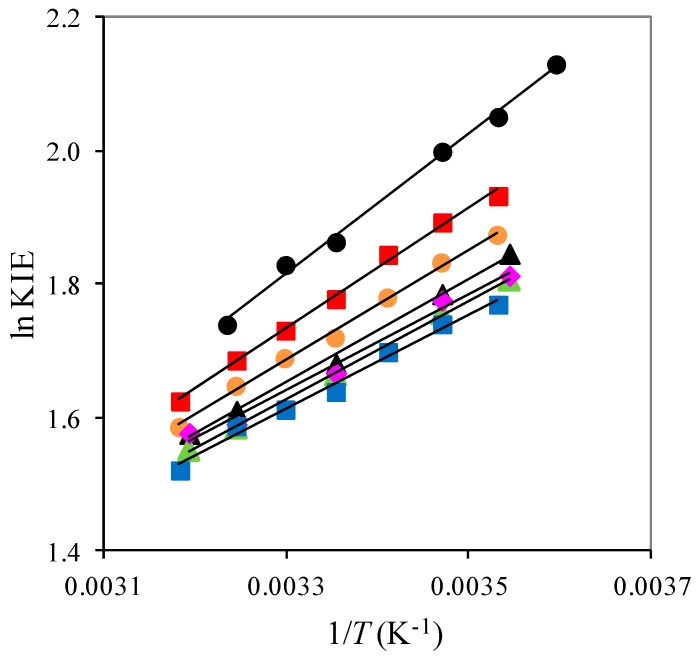
Temperature dependence of KIE in the reaction of ascorbate with hexacyanoferrate(III) ion in presence of different concentrations of quaternary ammonium salts in water. Key: green triangle, 0.005 M TMACl; black triangle, 0.005 M TPACl; magenta diamond, 0.005 M TBACl; blue square, 0.01 M TEACl; orange circle, 0.006 M PQCl_2_; red square, 0.1 M AChCl; black circle, 0.1 M BTMACl. Abbreviations as noted in Table 1.

**Figure 4:**
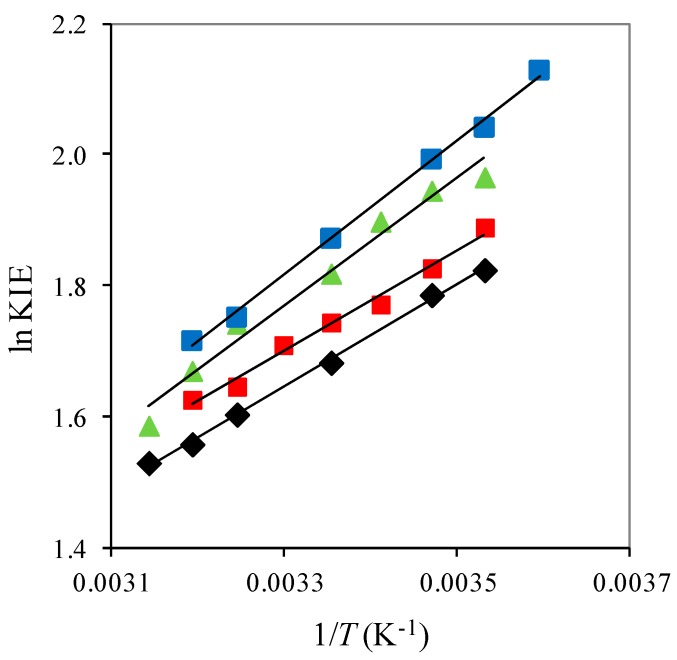
Temperature dependence of KIE in the reaction of ascorbate with hexacyanoferrate(III) ion in different organic cosolvent—water mixtures. Key: black diamond, 1,4-diox:water = 0.05:0.95 *v*/*v*; green triangle, 1,4-diox:water = 0.10:0.90 *v*/*v*; blue square, MeCN:water = 0.10:0.90 *v*/*v*; red circle, EtOH:water = 0.10:0.90 *v*/*v*. Abbreviations as noted in Table 1.

The kinetic isotope effects in the PCET reaction (Scheme 1) observed on addition of only small amounts of inert solutes here the partially hydrophobic cosolvent or quaternary ammonium ions in the water reaction solution increased unexpectedly with regard to KIE observed in the “neat” water solution. Thus, the surprising increase of the KIE has been observed already in the presence of millimolar concentrations of quaternary ammonium ions in the water reaction solution and similarly when the molar fraction of the cosolvents in the solution was of the order of 0.01 (see Table 1). The observed KIE are strongly temperature dependent (Figure 1, Figure 2, Figure 3 and Figure 4).

**Figure 5:**
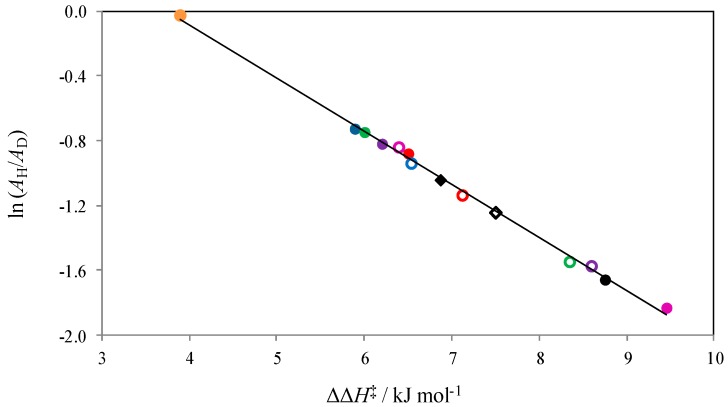
Correlation between ΔΔ*H*^‡^ (D,H) and ln *A*_H_/*A*_D_. Key: orange circle, neat water [35]; violet circle, 0.005 M TMACl; blue circle, 0.01 M TEACl; magenta circle, 0.1 M TEACl; red circle, 0.005 M TPACl; green circle, 0.005 M TBACl; black circle, 0.1 M BTMACl; hollow black diamond, 0.1 M AChCl; black diamond, 0.006 PQCl_2_; hollow blue circle, 1,4-dioxane:water = 0.05:0.95 *v*/*v*; hollow green circle, 1,4-dioxane:water = 0.1:0.9 *v*/*v*; hollow red circle, MeCN:water = 0.05:0.95 *v*/*v*; hollow violet circle, MeCN:water = 0.1:0.9 *v*/*v*; hollow magenta circle, EtOH:water = 0.1:0.9 *v*/*v*. Abbreviations as noted in Table 1.

The isotopic ratio of Arrhenius prefactors *A*_H_/*A*_D_ typical of the over-the-barrier reaction regime and the isotopic difference of Arrhenius activation energies *E*_a_^D^–*E*_a_^H^ (isotopic difference in activation enthalpies ΔΔ*H*^‡^) below the semiclassical limit have been observed in the investigated ascorbate PCET reaction performed in “neat” water. But the addition of small amounts of partially hydrophobic cosolvent or quaternary ammonium ions lead to the values of the isotopic ratios of Arrhenius prefactors *A*_H_/*A*_D_ well beyond the semiclassical limits [36,37,38,39,40] of 0.5–1.4 and to isotopic differences *E*_a_^D^–*E*_a_^H^ in the Arrhenius activation energies (or activation enthalpies ΔΔ*H*^‡^ (D,H) between D_2_O and H_2_O) which are greater than the semiclassical value of 5.1 kJ/mol for dissociation of O-H bond and are indicative of hydrogen tunnelling in the reaction (Table 1). Strongly linear correlation between the isotopic differences in the activation enthalpies ΔΔ*H*^‡^ (D,H) and ln *A*_H_/*A*_D_ (or the isotopic differences in entropies ΔΔ*S*^‡^ (D,H)) has been obtained for all the investigated systems (Figure 5).

Remarkably, the points for neat water and dioxane and acetonitrile and ethanol containing water solutions fall on the same straight line with quarternary ammonium ion solutions which strongly suggest that the process leading to the obtained isotopic differences in the activation enthalpies and entropies should be in all the cases, that of organic cosolvents as well as quaternary ammonium ions, of the same physical origin. Worth noting, the relationship obtained is not the consequence of correlation between Δ*H*^‡^ and Δ*S*^‡^ (enthalpy-entropy compensation, see in Supplementary Materials) in the reaction.

The increase of KIE and the appearance of the tunnelling signatures observed on the addition of milimolar amounts of the partially hydrophobic solutes into the water reaction PCET system seems to be highly surprising and unexpected; it should be unlikely that these milimolar concentrations of the inert solutes lead to any significant change of the bulk solvent properties which would eventually result in changes of the KIE.

Furtherly, taking into account the specificity of PCET reactions, as the various inert partially hydrophobic solutes, such as dioxane, acetonitrile, ethanol, quaternary ammonium ions etc., (see Table 1) were added into water reaction solution, one could doubt that these solutes will influence the KIE in the PCET reaction. Namely, the question might be whether there are significant changes of the reaction driving force Δ*G*° or the DAD due to any related effect of the solutes added. In accord with the theoretical treatments [16,17,22,34,35] related to PCET reactions, KIE accompanying H-transfer in the PCET processes will depend on the equilibrium proton donor-acceptor distance, the effective frequency Ω of donor-acceptor mode (R-mode), and driving force Δ*G*°. Briefly, KIE in a PCET reaction [16,17,24,34,35] will increase when the equilibrium proton DAD increases, when the effective frequency Ω of donor-acceptor mode (R-mode) increases, and when driving force, |Δ*G*°| decreases approaching a maximum near Δ*G*° ≈ 0.

However, the unexpected increase of KIE observed here on the addition of organic cosolvents into water reaction solution cannot be accounted neither for changes of the reaction driving force Δ*G*° nor changes in the proton donor-acceptor distance in the case. Certainly, driving force in our reaction should increase on addition of organic cosolvent [41] (since the p*K*_a_ of ascorbate monoanion increases and *E*°’ of hexacyanoferrate(III) decreases, on addition of organic cosolvents, see in Supplementary Materials); therefore, a decrease in the KIE due to the change in Δ*G*° should be expected but the opposite has been observed.

Furthermore, although only a subtle change in the DAD could lead to a change in KIE, it seems to be quite unlikely that the presence of one molecule of dioxane per hundred molecules of water in the system gives rise to a change of DAD that could be relevant for the effects observed. Moreover, as the proton DAD in the case of dioxane could be more increased with the decrease in solvent polarity (due to electrostatic repulsion of the negatively charged reactants, the ascorbate monoanion and hexacyanoferrate(III) anion) than in the case of acetonitrile, expectation would be that the addition of much more polar acetonitrile solvent into water reaction solution should lead to a smaller change in the KIE than the addition of much less polar dioxane. However, this is not what is found; essentially the same changes of KIE in the cases of dioxane and acetonitrile have been observed (Table 1).

The surprising increase of KIE in the investigated PCET reaction caused by the presence of only millimolar concentrations of quaternary ammonium ions could be ascribed neither to changes of DAD in the transition configuration nor to changes of driving force of reaction. The formation of ion pairs between the quaternary ammonium ions and hexacyanoferrate(III) ions might be taken into account [42] (see in Supplementary Materials). However, the proton DAD should decrease in the case. (There should be less electrostatic repulsion between the reactants than in the former case of unpaired hexacyanoferrate(III) ion). Consequently, expectation will be a decrease of KIE. In contrast, an increase of KIE has been observed in all the cases where quaternary ammonium ions were involved. In addition, a small change of reaction driving force caused by the ion pairing could be expected [37]. However, these small changes of driving force would not alter KIE in the process [17,31,34]. Finally, the fact that the isotopic differences ΔΔ*H*^‡^ (D,H) and the ratios ln *A*_H_/*A*_D_ are very well fitted into the same correlation in all the cases examined (Figure 5), including the very different partial hydrophobic substrates, can also be seen as a confirmation that the possible changes of KIE because of an influence to driving force or DAD caused by the solutes added should be insignificant in the cases; it should be highly unlikely that the correlation comprising such a number of experimentally determined parameters would be only a coincidence.

The hydrogen tunnelling observed as well as the modulation of hydrogen tunnelling in the PCET process induced by the small quantities of the quaternary ammonium ions or the organic cosolvent (as evidenced from the observed temperature dependence of KIE) will not be considered in the context of Bell tunnelling correction model [36] since the upper limit set by the model is ~6 kJ mol^−1^ for the ΔΔ*H*^‡^ (D,H) value in H-transfer process; the experimentally obtained values of the ΔΔ*H*^‡^ (D,H) in all the cases investigated here are greater than this figure. In addition, the increase in the barrier height (ΔΔ*G*^‡^) on going from “neat” water to 0.05:0.95 *v*/*v* dioxane—water is only ~1 kJ mol^−1^; moreover, in the cases of 0.01 mol dm^−3^ TEACl and 0.1 mol dm^−3^ BTMACl added, the reaction barrier decreases for 2 kJ mol^−1^ and ~4 kJ mol^−1^, respectively (see in Supplementary Materials). A decrease of the reaction barrier is expected to favour the over-the-barrier process; hence, one would not expect an increase of tunnelling contribution observed in the cases. Worth noting again, the tunnelling contribution can be seen from the Arrhenius equation where KIE is expressed as ln *k*_H_/*k*_D_ = ΔΔ*H*^‡^ (D,H)/RT + ln (*A*_H_/*A*_D_) (cf. Table 1) as in the over-the-barrier process ln (*A*_H_/*A*_D_) should be near the zero value and ΔΔ*H*^‡^ (D,H) should amount ~5 kJ mol^-1^.

More insight, however, can be supplied taking into account the dynamical features of the system, the approach common in the context of models related to the coupling of fast dynamics to the chemical step in enzymatic proton transfers [23,24,25,26,27,28,29,30,31,32,33] where dynamical aspects in optimizing the inter-nuclear distance (the hydrogen DAD) through sampling of tunnelling ready configurations and the consequent facilitating of hydrogen tunnelling are taken into regard. This means that the model takes into regard fast vibrational modes with some degree of motions along the reaction coordinate that can lead to hydrogen DAD compression on the timescale of barrier crossing, increasing thus the absolute nuclear quantum tunnelling probability [25,26,27]. Importantly, the theoretical treatments of PCET reactions also involve the DAD oscillations through the effective frequency Ω of donor-acceptor mode (R-mode). Consequently, our explanation of the observed tunnelling phenomena relies on the dynamical picture of the process where the involvement of collective and excitonic vibrational dynamics of the water reaction environment coupled with the dynamics of transition configuration (see below) should be taken into account in the case.

### 2.2. The Water Vibrational Dynamics and Hydrogen Tunnelling in the Reaction

Recent ultrafast femtosecond 2D IR spectroscopy experimental studies and theory established highly coupled and collective nature of water vibrations [1,2,3,4,5,6,7,8,9,10]. These dynamics are determined by the anharmonic nuclear potential energy surfaces of the extended hydrogen-bond network in liquid water where intermolecular couplings comparable to intramolecular couplings enable strong mixing between high and low frequency modes. This interplay of different high and low-frequency modes evolving in a strong yet fluctuating hydrogen bond network in the liquid water lead to the wealth of ultrafast vibrational dynamics. The OH stretching vibration of water cannot be described simply as local bond stretching or symmetric/asymmetric vibrations, but rather as a delocalized vibration, or vibrational exciton that involves coherent motion spanning up to six water molecules [1,2,4,5,6,7]. There is evidence [4] also that 2–7 water molecules are involved in the case of delocalized bend vibrations and that there exists strong stretch–bend coupling; it was demonstrated that the vibrational motions in liquid water have strongly mixed intra- and intermolecular character with contributions from the OH stretch, the H_2_O bend and the intermolecular vibrations [1,2,3,4].

Furthermore, solute stretches can show significant mixing with the water OH stretches if there is strong ion-water H-bonding; the ion and water modes can be intimately correlated in the hydration, as is established, for example, in the case of aqueous carbonate [3]. It is also proposed that the excitonic nature of the water vibrations, where the vibrational motions are delocalized over multiple water molecules, is an important factor in describing ion hydration.

**Figure 6:**
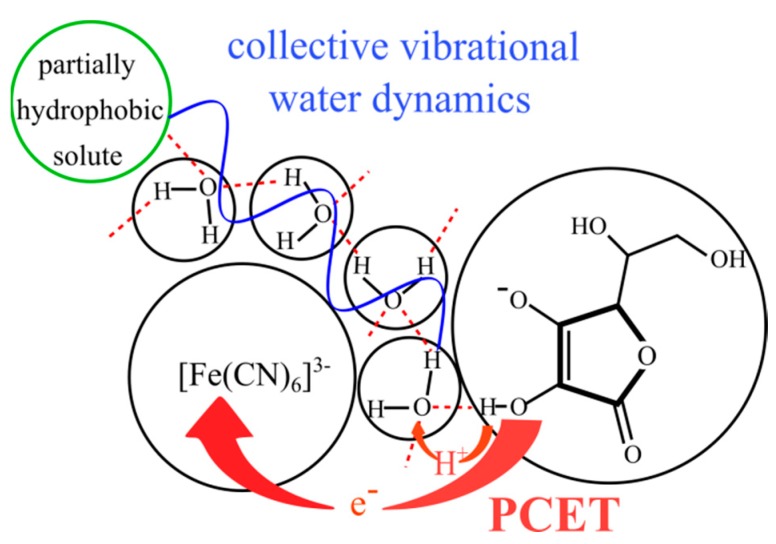
Schematic representation of transition configuration in the PCET ascorbate interaction with hexacyanoferrate(III) ion.

The consideration of possible influence of vibrational water dynamics on the hydrogen tunneling observed in the case should therefore take into regard the vibrational coupling between water and the partners constituting the transition reaction configuration surrounded by the aqueous solvation shell in the concerted PCET reaction (Figure 6). Importantly, the transition configuration in the PCET ascorbate interaction with hexacyanoferrate(III) involves the water molecule found between the redox partners, as confirmed earlier by the studies of proton inventory and KIE in the presence of acetate ions [19,20,21]. The proton transfers from 2-OH of ascorbate to this water molecule in the rate-controlling step of the PCET process. Hence, expectation should be that this water molecule constituting the transition configuration is hydrogen bonded with OH of the ascorbate monoanion. It should also be expected this water molecule to be hydrogen bonded with proximate water molecules from the aqueous solvation shell of the transition reaction configuration.

Finally, there is no doubt that the donor-acceptor motions (DAD distance fluctuations) can be of fundamental importance to achieve the configuration suitable for hydrogen tunnelling (tunnelling ready configuration) in the hydrogen transfer process; the effective barrier width (corresponding to the DAD optimized through sampling of tunnelling-ready configurations) is critical for the degree of nuclear quantum tunnelling in the case [25,26,27].

Collectively, given (1) that the water molecule found in the transition configuration can be hydrogen bonded and intertwined with aqueous solvation shell water molecules, and (2) taking into account the collective and intermolecular nature of vibrational dynamics of water as revealed by the ultrafast 2D IR spectroscopy and theory, and (3) taking into regard the role and importance of the DAD oscillations to the hydrogen tunneling probability, and finally, (4) given the ability of water molecule hydrogen-bonded to the solute to enter into motion correlated with the solute vibrations, it seems reasonably to conclude that the vibrational water dynamics can influence the extent of hydrogen tunnelling in the reaction investigated. This means that the collective and excitonic water vibrations can be correlated with the donor-acceptor motions along the reaction coordinate; then, optimization of DAD through sampling leads to an increase of absolute nuclear quantum tunnelling probability in the process [25,26,27]. Since the acceptor water molecule can be involved in the water hydrogen bonded network, this picture of DAD optimization should take into account the strongly mixed intra- and intermolecular collective character of water vibrations where the mixing of stretch, bend and intermolecular motions take place [1,2,3,4].

To this end, the connection of the partially hydrophobic molecules in the water solution and the tunneling modulation observed ought to be considered. It is well known that the presence of the partially hydrophobic solutes can lead to changes in vibrational dynamics of water due to, for example, C-H···O interactions between water and the solute molecules. These changes are already seen in the (linear) infrared absorption spectra [43,44,45]. Thus, in the dioxane-water system, the 3585 and 3505 cm^−1^ peaks, that are blue-shifted from that of pure water spectrum are attributed to vibrational bands of water molecules that share hydrogen bonding with 1,4-dioxane [44]. Similar interactions have been found in the cases of 1,3 dioxane [46], acetonitrile [47], tert butyl alcohol [48] and quaternary ammonium ions [49,50]. It could be added, the C-H···O interactions are considered theoretically to have fundamental properties of hydrogen bond [51]. Consequently it should be expected that the hydrogen bonding of partial hydrophobe with water molecules can change the dynamics of water i.e., the collective intermolecular excitonic dynamics in the case.

Therefore, expectation is that the presence of partially hydrophobic solutes leads to changes in vibrational dynamics of water that would be reflected in hydrogen tunneling modulation in the case. As the other system properties remain essentially unchanged, the only relevant changes induced by the addition of partially hydrophobic molecules appear to be those related to the water dynamics. Consequently, it seems reasonably to conclude that the observed modulation of hydrogen tunnelling in the presence of low concentrations of various partially hydrophobic solutes probably reflects the changes in the collective intermolecular vibrational dynamics of water due to hydrogen bonding interactions with the solutes; these dynamical changes would work through correlated motions of coupled water molecule and the transition configuration (involving the DAD oscillations along the reaction coordinate) in the process.

In other words, as the only relevant system property changed should be related to the water dynamics, the fact that the presence of small concentrations of the partially hydrophobic solutes can lead to the modulation of hydrogen tunnelling observed seems to be a compelling evidence in support of the interplay of intermolecular collective vibrational dynamics of water and the coupled transition configuration motions along the reaction coordinate in the process.

Experimental results summarized in Table 1 and Figure 5, suggest strongly that the above described interplay between the water vibrational dynamics and the dynamics of the coupled transition configuration indeed takes place in the process. The results obtained show the signatures of hydrogen tunneling to be modulated in the investigated process of ascorbate monoanion with hexacyanoferrate(III) ions in the presence of various partially hydrophobic molecules at low concentrations of the solutes. Importantly, strongly linear correlation of ΔΔ*H*^‡^ (D,H) and ln *A*_H_/*A*_D_ observed comprises all the cases investigated. Here, two findings are of key importance. First, the point for “neat” water falls on the same straight line (Figure 5) of the ΔΔ*H*^‡^ (D,H) and ln *A*_H_/*A*_D_ correlation pointing strongly to the common physical basis of the process both in neat water (which is critical to the excitonic nature of water vibrations) and in the dilute solutions. Secondly, the obtained relationship reveals the occurrence of hydrogen tunnelling modulation; obviously, the observed modulation of hydrogen tunnelling is connected with the presence of various partially hydrophobic solutes added. It could be added that seemingly, the isotopic ratio of the Arrhenius prefactors *A*_H_/*A*_D_ 0.97 (0.15) in the reaction in neat water solution that can be fitted within the semiclassical limits of 0.7–1.4 appears to be strange. However, this apparent contradiction can be resolved taking into regard that tunnelling could exist although *A*_H_/*A*_D_ can be close to their semiclassical values, as Stern and Weston demonstrated many years ago [52]. An example may be the reaction of ascorbate with 2,2,6,6-tetramethylpyperidine-1-oxyl radical where *A*_H_/*A*_D_ of 1.2 but KIE (H/D) of 31.1 was observed; obviously, there should be hydrogen tunnelling in the case [41].

## 3. Materials and Methods

All chemicals used were of analytical grade (Sigma-Aldrich, Fluka, Merck, Invitrogen). Tetramethylammonium, tetraethylammonium, tetrabutylammonium and tetrapropylammonium chloride were recrystallized and concentration of salt solutions was determined by potentiometric titration with silver nitrate. Heavy water (Aldrich, 99.9%) was twice distilled before use. All the solutions were prepared with doubly distilled, freshly boiled, carbon dioxide and oxygen-free (bubbled previously with 99.999% N_2_) water. The experimental procedures have been described previously [19,20,21,22]; it will only be briefly presented here (see also Supplementary Materials for more information). Pseudo-first order rate constants for the reaction of ascorbate with potassium hexacyanoferrate(III) (ascorbate concentration in excess) have been determined spectrophotometrically by monitoring the decrease of absorbance of potassium hexacyanoferrate(III) at 420 nm. An Ocean Optics S2000 spectrometer provided with a Quantum Northwest QPOD Temperature-Controlled Sample Compartment for Fiber Optic Spectroscopy was used throughout to collect spectral and absorbance-time data. Kinetic measurements were performed under carefully maintained temperature conditions (within the limits of ±0.1 °C). Pseudo-first order conditions were obtained using ascorbate concentration in 15 or 20-fold excess over the concentration of hexacyanoferrate(III) ion, typically 5 × 10^−4^ M Na-ascorbate and 5 × 10^−5^ M K_3_Fe(CN)_6_ in organic solvent-water mixtures and 3 × 10^−4^ M Na-ascorbate and 4 × 10^−5^ M K_3_Fe(CN)_6_ in presence of quaternary ammonium salts in water (unless otherwise noted), taking into account the 2:1 reaction stoichiometry. The second-order rate constants for the reaction have been calculated from the observed pseudo-first order rate constant using the concentration of ascorbate determined spectrophotometrically. KIE between ascorbate monoanion and its 2-OD derivative in the reaction were determined from the measurements of the corresponding rate constants in H_2_O and D_2_O solution.

## 4. Conclusions

The insights obtained from the study of hydrogen tunneling phenomena in the PCET interaction of ascorbate monoanion with hexacyanoferrate (III) ions performed in the fairly diluted water solutions containing the inert partially hydrophobic solutes can be viewed, at least tentatively, to be compelling evidence in support of an interplay of delocalized collective intermolecular vibrational dynamics of water correlated with vibrations of the coupled transition configuration, where the donor-acceptor oscillations, the motions being to some degree along the reaction coordinate, lead to an enhancing of probability of hydrogen tunnelling in the reaction.

The key experimental evidence obtained, the modulation of hydrogen tunneling and the correlation between the hydrogen tunnelling signatures, the isotopic differences in the activation enthalpies ΔΔ*H*^‡^ (D,H) and the isotopic Arrhenius prefactor ratios ln *A*_H_/*A*_D_ (or ΔΔ*S*^‡^ (D,H)), as revealed from the observed temperature dependence of the KIE in the PCET reaction of ascorbate monoanion with hexacyanoferrate(III) ions in the fairly diluted water solutions containing various partially hydrophobic solutes, pointed strongly to the common physical origin of the observed hydrogen tunnelling modulation and surprisingly increased KIE in all the systems studied. Since the only relevant system property varying in the cases of the fairly diluted water solutions applied can be related to the changes of water dynamics due to presence of hydrogen-bonded partially hydrophobic molecules of the inert solutes added, the results obtained seem to suggest strongly that the common cause of the tunneling phenomena observed can be traced in the interaction of delocalized collective intermolecular vibrational dynamics of water correlated with vibrations of the coupled transition configuration in the process.

The picture proposed here in the case of the ascorbate PCET reaction in diluted water solutions appears to be consistent with the experimental and theoretical findings related to the recent ultrafast two-dimensional infrared transient absorption and polarization anisotropy spectroscopies of water [1,2,3,4] where the studies suggest the collective intermolecular and excitonic character of the relevant vibrations of water hydrogen network and correlated motions between water and solutes when there is strong H-bonding involved. It should also be consistent with the notion that highly coupled and collective vibrational motions in water would have significant implications for the way in which aqueous chemical reactions that involve transition configurations containing hydrogen bonded water molecule occur [1,2,3,4]. Therefore, the obtained evidence will be seen as the experimental confirmation of the proposal of involvement of ultrafast vibrational water dynamics in activation processes.

Finally, the obtained results offer some new insights related to dynamics in water environments in contact with partially hydrophobic species. Although the effects of partially hydrophobic species described here should not be viewed in the context of hydrophobicity commonly considered to underpin self-assembling processes [53,54,55] the phenomenon of interplay between dynamics involving partial hydrophobes and hydrogen bonded water network and the PCET reaction system observed in the case will be of significance for many situations where the dynamics of water in contact with hydrophobic species could be relevant to a hydrogen transfer/tunnelling process.

## Figures and Tables

**Table 1:** Kinetic Isotope Effects, Isotopic Differences ΔΔ*H* ^‡^ (D,H) and Ratios *A*_H_/*A*_D_ in the Reaction of Ascorbate with Hexacyanoferrate(III) Ions (Scheme 1).

Solute Added ^a^	KIE ^b^	DD*H*^‡^/kJ mol^−1^	*A*_H_/*A*_D_
none (neat water ^c^)	4.60 (0.06)	3.9 (0.4)	0.97 (0.15)
1,4-diox:water = 0.05:0.95 *v*/*v*	5.42 (0.10)	6.5 (0.2)	0.39 (0.03)
1,4-diox:water = 0.10:0.90 *v*/*v*	6.17 (0.08)	8.4 (0.6)	0.21 (0.05)
MeCN:water = 0.05:0.95 *v*/*v* ^d^	5.61 (0.16)	7.1 (0.4)	0.32 (0.04)
MeCN:water = 0.10:0.90 *v*/*v*	6.50 (0.10)	8.6 (0.2)	0.21 (0.02)
EtOH:water = 0.10:0.90 *v*/*v*	5.72 (0.06)	6.4 (0.3)	0.43 (0.05)
*c*(TMACl) = 0.005 mol dm^−3^	5.28 (0.03)	6.2 (0.4)	0.44 (0.03)
*c*(TPACl) = 0.005 mol dm^−3^	5.38 (0.05)	6.5 (0.4)	0.41 (0.04)
*c*(TBACl) = 0.005 mol dm^−3^	5.28 (0.08)	6.0 (0.4)	0.48 (0.07)
*c*(TEACl) = 0.01 mol dm^−3^	5.15 (0.02)	5.9 (0.3)	0.48 (0.06)
*c*(TEACl) = 0.1 mol dm^−3 e^	7.25 (0.02)	9.5 (0.2)	0.16 (0.01)
*c*(BTMACl) = 0.1 mol dm^−3^	6.46 (0.09) ^e^	8.7 (0.2)	0.19 (0.02)
*c*(AChCl) = 0.1 mol dm^−3^	5.91 (0.16)	7.5 (0.3)	0.29 (0.03)
*c*(PQCl_2_) = 0.006 mol dm^−3^	5.57 (0.17)	6.9 (0.2)	0.35 (0.03)

^a^ Abbreviations are as follows: dioxane, (1,4-diox); acetonitrile, (MeCN); ethanol, (EtOH); tetramethylammonium chloride, (TMACl); tetraethylammonium chloride, (TEACl); tetrapropylammonium chloride, (TPACl); tetrabutylammonium chloride, (TBACl); benzyltrimethylammonium chloride, (BTMACl); acetylcholine chloride, (AChCl); paraquat dichloride hydrate, (PQCl_2_); ^b^ At 298K; ^c^ from ref. [19]; ^d^ from ref. [20]; ^e^ from ref. [21].

## Supplementary Materials

The following are available online, The Reaction of Ascorbate with Hexacyanoferrate(III) Ions; Experimental; The Temperature Dependence of KIE-s in the Reaction in the Presence of Cosolvent or Quaternary Ions Added; Thermochemistry; Ion Pairing in the Presence of Quaternary Ions.
