# Supplementary material for: Hydrogen Tunnelling as a Probe of the Involvement of Water Vibrational Dynamics in Aqueous Chemistry?
Source: Molecules. 2019 Dec 31;25(1):172. doi: 10.3390/molecules25010172 (PMC6983115; doi:10.3390/molecules25010172)
Supplement: Supplementary file 1 [file molecules-25-00172-s001.pdf]

Supplementary Material:

Hydrogen Tunnelling as a Probe of the Involvement of Water Vibrational Dynamics in Aqueous Chemistry?

Ana Karković Marković, Cvijeta Jakobušić Brala*, Viktor Pilepić and Stanko Uršić*

Faculty of Pharmacy and Biochemistry, University of Zagreb, A. Kovačića 1, 10 000 Zagreb, Croatia; cjakobus@pharma.hr

1. The Reaction of Ascorbate with Hexacyanoferrate(III) Ions

The investigated process is the known* interaction of monoascorbate anion with hexacyanoferrate(III) (Scheme S1).

Scheme S1

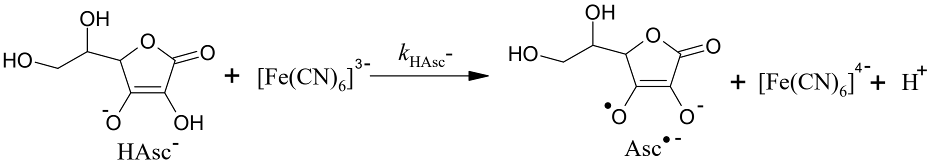

Ascorbate reduces hexacyanoferrate(III), giving hexacyanoferrate(II) and dehydroascorbic acid. The overall stoichiometry of the reaction is 1:2 (ascorbate:hexacyanoferrate(III)). The ascorbyl radical anion formed in the relatively slow first step (Scheme S1) will reduce another $[Fe(CN)_6]^{3-}$ in the fast subsequent reaction:

$$Asc^{\bullet -} + Fe(CN)_6^{3-} \longrightarrow Fe(CN)_6^{4-} + \text{dehydroascorbic acid}$$

The rate law for the reaction is:

$$rate = -d\,[Fe(CN)_6^{3-}]/d\,t = 2k_{HAsc^-}\,[HAsc^-]\,[Fe(CN)_6^{3-}]$$

$$k_{obs} = 2k_{HAsc^-}[HAsc^-]$$

The rate parameters for the reaction of ascorbate with hexacyanoferrate(III) (Scheme S1) have been obtained from the pseudo-first order rate constants (k_{obs}). Pseudo-first order rate constants have been determined spectrophotometrically by monitoring the decrease

of absorbance of potassium hexacyanoferrate(III) at 420 nm (constant ascorbate concentration, see below).

---

* references 18-23 in manuscript

2. Experimental

Materials. Sodium *L*-ascorbate (≥ 99%), *L* -asorbic acid, potassium hexacyanoferrate(III), EDTA (ethylenediaminetetraacetate) disodium salt, tetramethylammonium chloride, tetraethylammonium chloride, benzyltrimethylammonium chloride, tetrapropylammonium chloride, tetrabutylammonium chloride, acetylcholine chloride, paraquat dichloride hydrate, heavy water (99.9% D), 1,4-dioxane, acetonitrile, ethanol, ethanol (D), methanol, acetone, diethyl ether and perchloric acid (70%) were analytical grade (Sigma-Aldrich, Fluka, Merck, Invitrogen). Tetramethylammonium chloride was recrystallized from methanol and acetone, tetraethylammonium chloride and acetylcholine chloride from ethanol and diethyl ether, tetrabutyl and tetrapropyl ammonium chloride from acetone. Concentration of salt solutions was determined by potentiometric titration with silver nitrate (Poch, *p.a.*). Heavy water (Aldrich, 99.9%) was twice distilled before use. All the solutions were prepared with doubly distilled, freshly boiled, carbon dioxide and oxygen-free (bubbled previously with 99.999% N_2) water.

Methods. Kinetic Measurements. Pseudo-first order rate constants for the reaction of ascorbate with potassium hexacyanoferrate(III) have been determined spectrophotometrically by monitoring the decrease of absorbance of potassium hexacyanoferrate(III) at 420 nm. An Ocean Optics S2000 spectrometer provided with a Quantum Northwest QPOD Temperature-Controlled Sample Compartment for Fiber Optic Spectroscopy was used throughout to collect spectral and absorbance-time data. Kinetic measurements were performed under carefully maintained temperature conditions (within the limits of ± 0.1°C). In a typical kinetic run, at least 200 pairs of

absorbance-time data were collected and fitted to the common least-square algorithm. At least three to four observed pseudo-first rate constants were always used to calculate the corresponding rate parameters under the specified reaction conditions. The measurements were performed under the pseudo-first order conditions, taking ascorbate concentration to be typically 15 or 20-fold in excess, (taking into regard the 2:1 reaction stoichiometry) over the concentration of hexacyanoferrate(III) ion. Very good pseudo-first order kinetics were obtained throughout. The second-order rate constants for the reaction have been calculated from the observed pseudo-first order rate constant using the concentration of ascorbate determined spectroscopically (see below) in the calculation. Furthermore, essentially the same rate constants (within the limits of the experimental error) were obtained when the ascorbate excess was 12-fold (experiments with benzyltrimethylammonium chloride and acetylcholine chloride) as in the case of 20-fold excess of ascorbate.

The conditions and procedure of the kinetic measurements involved also the following: the pseudo-first order rate constants were determined typically at neutral pH, 5.6 – 6.6, in the presence of EDTA disodium salt ($5 \cdot 10^{-4}$ M). The 0.1 M stock solutions of ascorbate for kinetic measurements were prepared by dissolving ascorbate in deoxygenated water containing $5 \cdot 10^{-4}$ M EDTA disodium salt. Initially, reaction solution without potassium hexacyanoferrate(III) has been thermostatted for 20 minutes before the kinetics were started. The reaction has always been initiated by addition of appropriate solution (water or organic solvent-water) of hexacyanoferrate(III) ion into 1.8 mL of reaction solution.

Kinetic isotope effects between ascorbate monoanion and its 2-OD derivative in the reaction were determined from the measurements of the corresponding rate constants in H_2O and D_2O solution. The H_2O content in all the experiments with D_2O was always in range of 0.1 – 0.5 % (D_2O used was 99.9%). The reported values of KIE-s are corrected for

the H_2O in D_2O content. The observed KIE-s differ from the corrected ones only slightly (within 1.5 – 2.5%).

The concentration of ascorbate in the reaction mixture has been typically determined by measuring the absorbance of ascorbate at 294 or 300 nm before each kinetic run. The molar absorption coefficients of ascorbate at 294 and 300 nm have been determined, at temperatures from 5.0 to 45.0 °C ± 0.1 °C, in each reaction solvent (organic solvent-water/heavy water mixture or water/heavy water with added salts) used. Water and heavy water were doubly distilled, freshly boiled, carbon dioxide and oxygen-free (bubbled previously with 99.999% N_2). Under employed conditions (5×10^{-4} M sodium ascorbate solution), the only relevant molecular ascorbate species is the ascorbate monoanion ($HAsc^-$). Additionally, ascorbate monoanion concentration in experiments in acidic reaction medium (experiments with $PQCl_2$) has been determined with Multi-Component Quantification Analysis using HP ChemStation UV-Vis Software (A.08.03) from the UV-Vis spectra of the reaction mixture before each kinetic run and UV-Vis spectra of ascorbate and ascorbic acid of known concentrations in water/heavy water as calibration standards in the appropriate wavelength range, usually from 220 – 330 nm. The UV-Vis spectra of ascorbate and ascorbic acid used as calibration standards have been measured in conditions where the only relevant species is ascorbate monoanion or ascorbic acid, respectively. Using this procedure, a painstaking determination of relatively small changes of pK_a values due to variation in ionic strength was avoided.

3. The Temperature Dependence of KIE-s in the Reaction in the Presence of Cosolvent or Quaternary Ions Added

Table S1. Rate Constants and Corresponding Kinetic Isotope Effects for the Reaction of Ascorbate with Hexacyanoferrate(III) ion at Various Temperatures in 1,4-Dioxane-Water Solvent Mixture (0.05 : 0.95 v/v), at Ionic Strength I = 0.0023.

t/°C	**H_2O** k_{HAsc^-}/$M^{-1}s^{-1}$	**D_2O** k_{HAsc^-}/$M^{-1}s^{-1}$	KIE
10.0	28.8 (0.3)	4.61 (0.07)	6.25 (0.12)
15.0	34.0 (0.2)	5.66 (0.06)	6.01 (0.07)
25.0	47.3 (0.5)	8.73 (0.14)	5.42 (0.10)*
35.0	63.6 (0.5)	12.7 (0.2)	5.01 (0.10)
40.0	73.2 (0.3)	15.3 (0.1)	4.77 (0.05)
45.0	83.9 (0.4)	18.1 (0.2)	4.64 (0.07)

$5 \cdot 10^{-4}$ M Na-ascorbate, $5 \cdot 10^{-5}$ M $K_3Fe(CN)_6$, $5 \cdot 10^{-4}$ M Na_2EDTA
*The figure within this series; in good agreement with earlier one (determined at 298K only, reference 20 in manuscript: Karković, A.; Jakobušić Brala, C.; Pilepić, V.; Uršić, S. Solvent-Induced Hydrogen Tunnelling in Ascorbate Proton-Coupled Electron Transfers. *Tetrahedron Lett.* **2011**, *52* (15), 1757–1761.)

Table S2. Rate Constants and Corresponding Kinetic Isotope Effects for the Reaction of Ascorbate with Hexacyanoferrate(III) ion at Various Temperatures in 1,4-Dioxane-Water Solvent Mixture (0.1 : 0.9 v/v), at Ionic Strength I = 0.0023.

t/°C	**H_2O** k_{HAsc^-}/$M^{-1}s^{-1}$	**D_2O** k_{HAsc^-}/$M^{-1}s^{-1}$	KIE
10.0	18.9 (0.1)	2.65 (0.01)	7.13 (0.05)
15.0	23.3 (0.4)	3.33 (0.05)	7.00 (0.16)
20.0	27.9 (0.7)	4.18 (0.04)	6.67 (0.19)
25.0	33.5 (0.3)	5.43 (0.05)	6.17 (0.08)
35.0	45.4 (0.7)	7.96 (0.08)	5.70 (0.10)
40.0	53.5 (0.5)	10.34 (0.07)	5.17 (0.06)
45.0	60.8 (0.8)	12.45 (0.35)	4.88 (0.15)

$5 \cdot 10^{-4}$ M Na-ascorbate, $5 \cdot 10^{-5}$ M $K_3Fe(CN)_6$, $5 \cdot 10^{-4}$ M Na_2EDTA

Table S3. Rate Constants and Corresponding Kinetic Isotope Effects for the Reaction of Ascorbate with Hexacyanoferrate(III) Ion at Various Temperatures in MeCN-Water Solvent Mixture (0.1 : 0.9 v/v), at Ionic Strength I = 0.0023.

t/°C	H_2O k_{HAsc^-}/$M^{-1}s^{-1}$	D_2O k_{HAsc^-}/$M^{-1}s^{-1}$	KIE
5.0	22.3 (0.1)	2.66 (0.03)	8.38 (0.11)
10.0	26.5 (0.1)	3.43 (0.08)	7.73 (0.19)
15.0	31.1 (0.1)	4.24 (0.02)	7.33 (0.03)
25.0	43.1 (0.6)	6.63 (0.02)	6.50 (0.10)
35.0	57.2 (0.2)	9.93 (0.01)	5.76 (0.02)
40.0	66.6 (0.1)	12.00 (0.10)	5.55 (0.04)

$5 \cdot 10^{-4}$ M Na-ascorbate, $5 \cdot 10^{-5}$ M $K_3Fe(CN)_6$, $5 \cdot 10^{-4}$ M Na_2EDTA

Table S4. Rate constants and corresponding kinetic isotope effects for the reaction of ascorbate with hexacyanoferrate(III) ion at various temperatures in ethanol-water solvent mixture (0.1 : 0.9 v/v), at ionic strength I = 0.0023.

t/°C	**H_2O**<br>k_{HAsc^-}/$M^{-1}s^{-1}$	**D_2O**<br>k_{HAsc^-}/$M^{-1}s^{-1}$	KIE
10.0	29.3 (0.6)	4.44 (0.05)	6.60 (0.16)
15.0	34.1 (0.3)	5.49 (0.12)	6.21 (0.15)
20.0	39.7 (0.1)	6.75 (0.13)	5.88 (0.12)
25.0	46.9 (0.4)	8.20 (0.05)	5.72 (0.06)
30.0	53.6 (0.3)	9.70 (0.06)	5.53 (0.05)
35.0	61.4 (1.1)	11.83 (0.04)	5.20 (0.10)
40.0	70.8 (0.8)	13.92 (0.29)	5.09 (0.12)

$5\cdot10^{-4}$ M Na-ascorbate, $5\cdot10^{-5}$ M $K_3Fe(CN)_6$, $5\cdot10^{-4}$ M Na_2EDTA

Table S5. Rate constants and Corresponding Kinetic Isotope Effects for the Reaction of Ascorbate with Hexacyanoferrate(III) Ion at Various Temperatures in Water, in Presence of 0.005 M Tetramethylammonium Chloride (TMACl).

T/°C	**H_2O**<br>k_{HAsc^-}/$M^{-1}s^{-1}$	**D_2O**<br>k_{HAsc^-}/$M^{-1}s^{-1}$	KIE
9.0	69.1 (0.6)	11.4 (0.2)	6.06 (0.12)
15.0	81.2 (0.6)	14.0 (0.1)	5.80 (0.05)
25.0	106.2 (0.6)	20.1 (0.0)	5.28 (0.03)
35.0	136.1 (0.5)	27.9 (0.0)	4.88 (0.02)
40.0	153.6 (1.3)	32.6 (0.3)	4.71 (0.06)

$3\cdot10^{-4}$ M Na-ascorbate, $4\cdot10^{-5}$ M $K_3Fe(CN)_6$, $5\cdot10^{-4}$ M Na_2EDTA

Table S6. Rate Constants and Corresponding Kinetic Isotope Effects for the Reaction of Ascorbate with Hexacyanoferrate(III) Ion at Various Temperatures in Water, in Presence of 0.005 M Tetrapropylammonium Chloride (TPACl).

t/°C	**H_2O**<br>k_{HAsc^-}/$M^{-1}s^{-1}$	**D_2O**<br>k_{HAsc^-}/$M^{-1}s^{-1}$	KIE
9.0	48.5 (0.6)	7.7 (0.0)	6.30 (0.08)
15.0	58.9 (0.5)	9.9 (0.1)	5.95 (0.07)
25.0	80.2 (0.6)	14.9 (0.1)	5.38 (0.05)
35.0	106.7 (1.2)	21.3 (0.2)	5.01 (0.08)
40.0	122.3 (1.0)	25.3 (0.3)	4.83 (0.07)

$3\cdot10^{-4}$ M Na-ascorbate, $4\cdot10^{-5}$ M $K_3Fe(CN)_6$, $5\cdot10^{-4}$ M Na_2EDTA

Table S7. Rate Constants and Corresponding Kinetic Isotope Effects for the Reaction of Ascorbate with Hexacyanoferrate(III) Ion at Various Temperatures in Water, in Presence of 0.005 M Tetrabutylammonium Chloride (TBACl).

t/°C	**H_2O**<br>k_{HAsc^-}/$M^{-1}s^{-1}$	**D_2O**<br>k_{HAsc^-}/$M^{-1}s^{-1}$	KIE
9.0	46.9 (0.3)	7.7 (0.0)	6.09 (0.05)
15.0	58.0 (0.5)	9.8 (0.2)	5.92 (0.11)
25.0	79.2 (1.1)	15.0 (0.1)	5.28 (0.08)
35.0	105.5 (1.2)	21.5 (0.1)	4.91 (0.06)
40.0	123.6 (1.0)	25.6 (0.1)	4.83 (0.05)

$3\cdot10^{-4}$ M Na-ascorbate, $4\cdot10^{-5}$ M $K_3Fe(CN)_6$, $5\cdot10^{-4}$ M Na_2EDTA

Table S8. Rate Constants and Corresponding Kinetic Isotope Effects for the Reaction of Ascorbate with Hexacyanoferrate(III) Ion at Various Temperatures in Water, in Presence of 0.01 M Tetraethylammonium Chloride (TEACl).

t/°C	**H_2O** k_{HAsc^-}/$M^{-1}s^{-1}$	**D_2O** k_{HAsc^-}/$M^{-1}s^{-1}$	KIE
10.0	72.2 (1.8)	12.3 (0.2)	5.87 (0.18)
15.0	83.6 (1.0)	14.7 (0.1)	5.69 (0.08)
20.0	94.9 (0.3)	17.4 (0.4)	5.45 (0.11)
25.0	108.7 (0.2)	21.1 (0.1)	5.15 (0.02)
30.0	123.4 (1.1)	24.6 (0.4)	5.02 (0.08)
35.0	142.3 (0.4)	29.1 (0.2)	4.89 (0.04)
41.0	160.8 (2.2)	35.3 (0.1)	4.56 (0.06)

$3\cdot10^{-4}$ M Na-ascorbate, $4\cdot10^{-5}$ M $K_3Fe(CN)_6$, $5\cdot10^{-4}$ M Na_2EDTA

Table S9. Rate Constants and Corresponding Kinetic Isotope Effects for the Reaction of Ascorbate with Hexacyanoferrate(III) Ion at Various Temperatures in Water, in Presence of 0.1 M Benzyltrimethylammonium Chloride (BTMACl).

t/°C	**H$_2$O** k_{HAsc^-}/M^{-1}s^{-1}	**D$_2$O** k_{HAsc^-}/M^{-1}s^{-1}	KIE
5.0	218 (2)	25.8 (0.1)	8.45 (0.08)
10.0	241 (3)	31.0 (1.0)	7.77 (0.26)
15.0	269 (6)	36.4 (0.3)	7.39 (0.17)
25.0	328 (3)	50.8 (0.6)	6.46 (0.09)*
30.0	368 (5)	59.1 (0.6)	6.23 (0.10)
36.0	409 (1)	71.8 (1.5)	5.70 (0.12)

$1.5\cdot10^{-4}$ M Na-ascorbate, $2.5\cdot10^{-5}$ M $K_3Fe(CN)_6$, $5\cdot10^{-4}$ M Na_2EDTA

* The figure within this series; in good agreement with earlier one (determined at 298K only, reference 21 in manuscript: Jakobušić Brala, C.; Karković, A.; Sajenko, I.; Pilepić, V.; Uršić, S. Sizeable Increase of Kinetic Isotope Effects and Tunnelling in Coupled Electron–Proton Transfers in Presence of the Quaternary Ions. PCET Processes and Hydrogen Tunnelling as a "Probe" for Structuring and Dynamical Phenomena in Water Solution. *Zeitschrift für Phys. Chemie* **2012**, *226* (1), 29–46.)

Table S10. Rate Constants and Corresponding Kinetic Isotope Effects for the Reaction of Ascorbate with Hexacyanoferrate(III) Ion at Various Temperatures in Water, in Presence of 0.1 M Acetylcholine Chloride (AChCl).

t/°C	**H$_2$O** k_{HAsc^-}/M^{-1}s^{-1}	**D$_2$O** k_{HAsc^-}/M^{-1}s^{-1}	KIE
10.0	188 (1)	27.2 (0.7)	6.91 (0.18)
15.0	212 (3)	31.9 (0.3)	6.65 (0.12)
20.0	244 (10)	38.6 (0.5)	6.32 (0.27)
25.0	276 (2)	46.7 (1.3)	5.91 (0.16)
30.0	310 (6)	54.9 (1.1)	5.65 (0.16)
35.0	348 (2)	64.5 (0.3)	5.40 (0.04)

41.0	397 (5)	78.2 (0.8)	5.08 (0.06)

1.5·10^{-4} M Na-ascorbate, 2.5·10^{-5} M $K_3Fe(CN)_6$, 5·10^{-4} M Na_2EDTA

Table S11. Rate Constants and Corresponding Kinetic Isotope Effects for the Reaction of Ascorbate with Hexacyanoferrate(III) Ion at Various Temperatures in Water, in Presence of 0.006 M Paraquat Dichloride Hydrate ($PQCl_2$).

t/°C	**H_2O** k_{HAsc^-}/M^{-1}s^{-1}	**D_2O** k_{HAsc^-}/M^{-1}s^{-1}	KIE
10.0	615 (14)	95 (2)	6.47 (0.20)
15.0	671 (20)	107 (3)	6.27 (0.21)
20.0	726 (2)	123 (2)	5.90 (0.11)
25.0	786 (20)	141 (2)	5.57 (0.17)
30.0	845 (22)	155 (3)	5.45 (0.17)
35.0	923 (16)	178 (3)	5.19 (0.12)
41.0	1001 (26)	205 (5)	4.88 (0.17)

3·10^{-4} M ascorbic acid, 4·10^{-5} M $K_3Fe(CN)_6$, 5·10^{-4} M Na_2EDTA, 5·10^{-4} M $HClO_4$ in H_2O (10^{-4} M $HClO_4$ in D_2O)

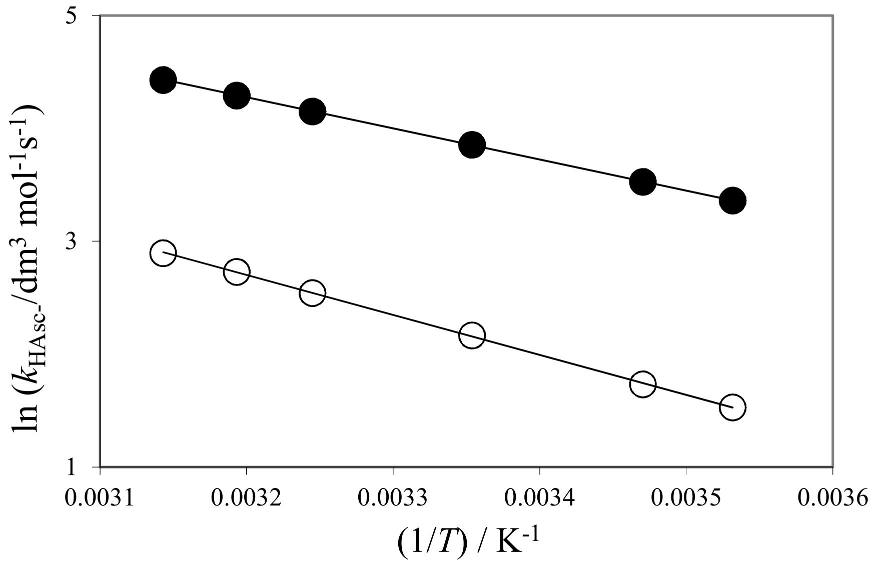

Figure S1. Arrhenius plot for the reaction of ascorbate and hexacyanoferrate(III) ion in 1,4-dioxane-H_2O solvent mixture (0.05 : 0.95 v/v) (●), r = 0.999 and 1,4-dioxane-D_2O solvent mixture (0.05 : 0.95 v/v) (○), r = 0.999, at ionic strength I = 0.0023. Data from Table S1.

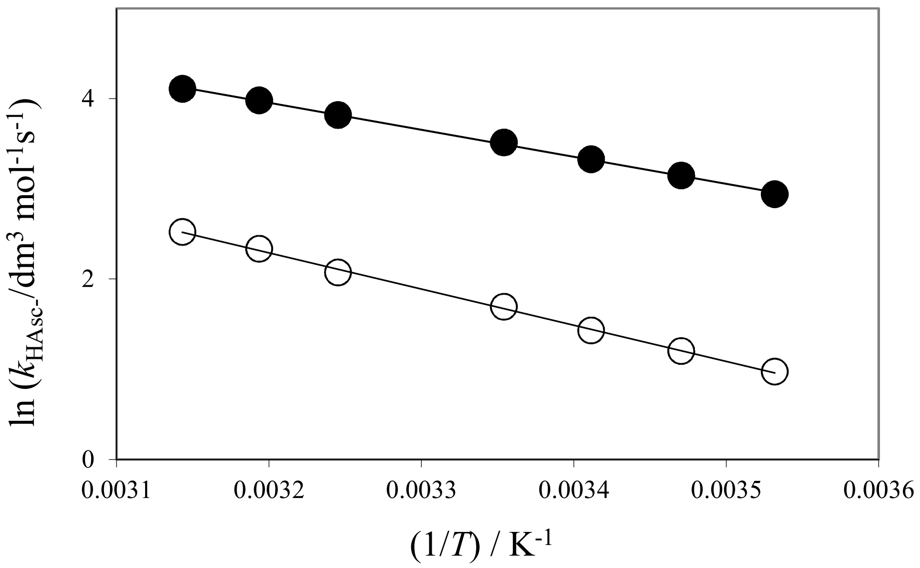

Figure S2. Arrhenius plot for the reaction of ascorbate and hexacyanoferrate(III) ion in 1,4-dioxane-H_2O solvent mixture (0.1 : 0.9 v/v) (●), r = 0.999 and 1,4-dioxane-D_2O solvent mixture (0.1 : 0.9 v/v) (○), r = 0.999, at ionic strength I = 0.0023. Data from Table S2.

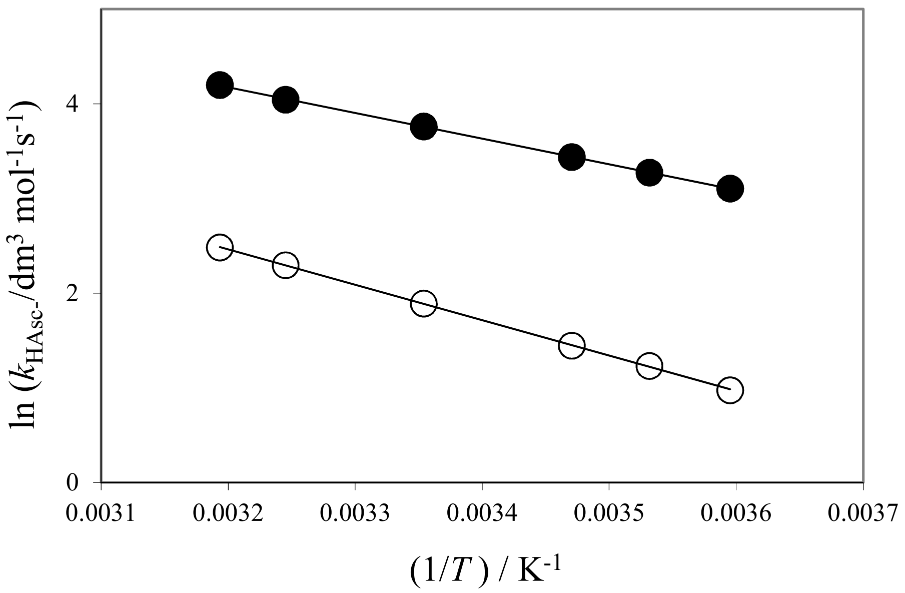

Figure S3. Arrhenius plot for the reaction of ascorbate and hexacyanoferrate(III) ion in MeCN-H_2O solvent mixture (0.1 : 0.9 v/v) (●), r = 0.999 and MeCN-D_2O solvent mixture (0.1 : 0.9 v/v) (○), r = 0.999, at ionic strength I = 0.0023. Data from Table S3.

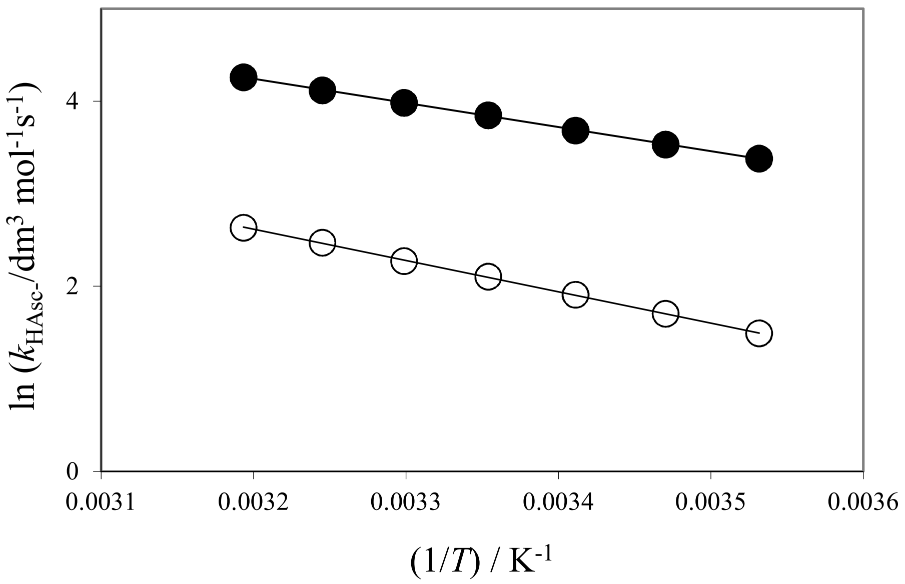

Figure S4. Arrhenius plot for the reaction of ascorbate and hexacyanoferrate(III) ion in EtOH-H_2O solvent mixture (0.1 : 0.9 v/v) (●), r = 0.999 and EtOD-D_2O solvent mixture (0.1 : 0.9 v/v) (○), r = 0.999, at ionic strength I = 0.0023. Data from Table S4.

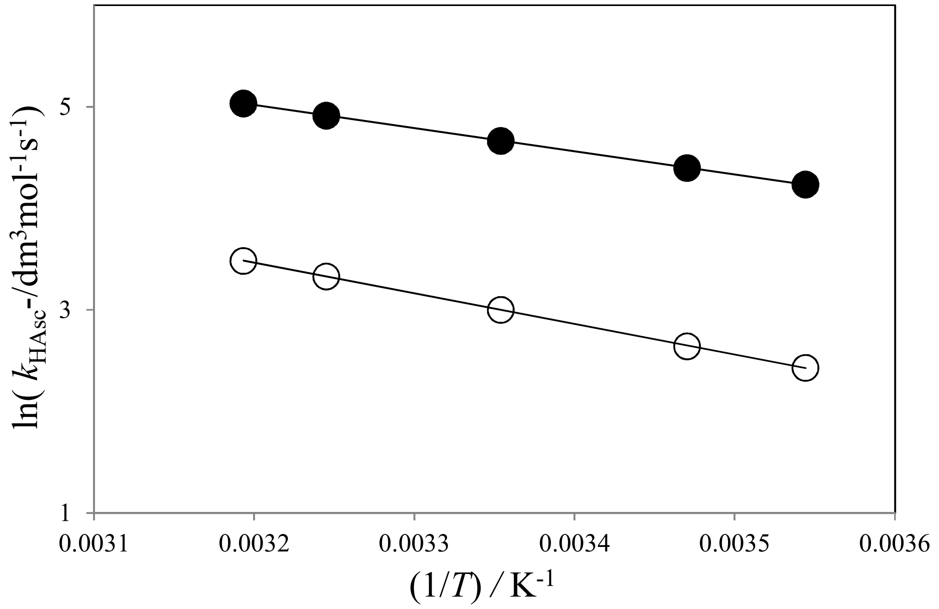

Figure S5. Arrhenius plot for the reaction of ascorbate and hexacyanoferrate(III) ion in presence of 0.005 M tetramethylammonium chloride (TMACl) in H_2O (●), r = 0.999 and D_2O (○), r = 0.999. Data from Table S5.

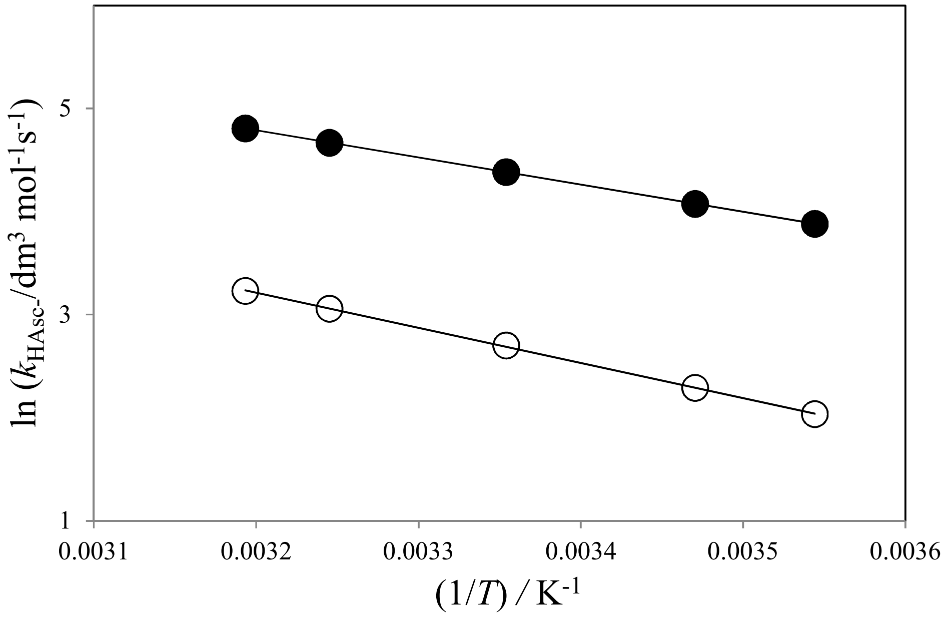

Figure S6. Arrhenius plot for the reaction of ascorbate and hexacyanoferrate(III) ion in presence of 0.005 M tetrapropylammonium chloride (TPACl) in H_2O (●), r = 0.999 and D_2O (○), r = 0.999. Data from Table S6.

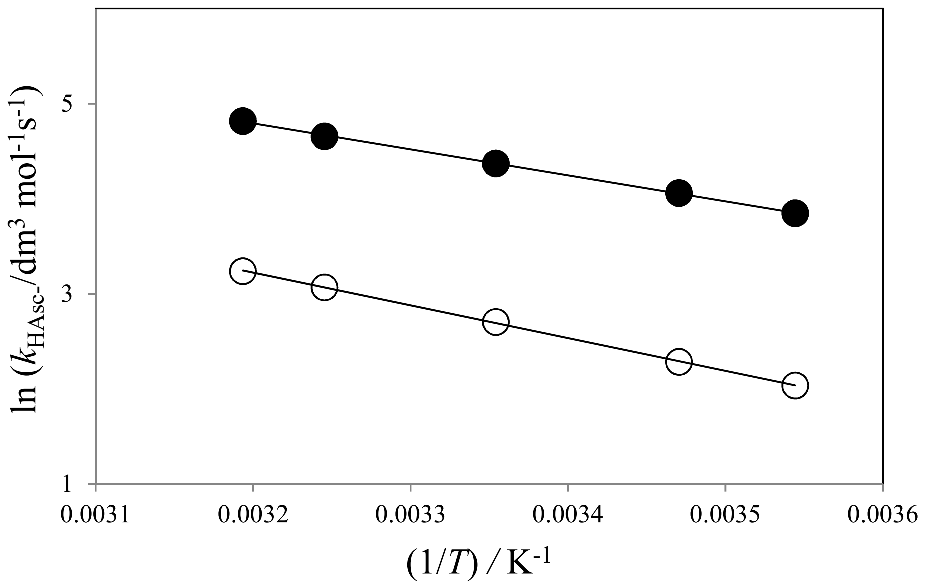

Figure S7. Arrhenius plot for the reaction of ascorbate and hexacyanoferrate(III) ion in presence of 0.005 M tetrabutylammonium chloride (TBACl) in H_2O (●), r = 0.999 and D_2O (○), r = 0.999. Data from Table S7.

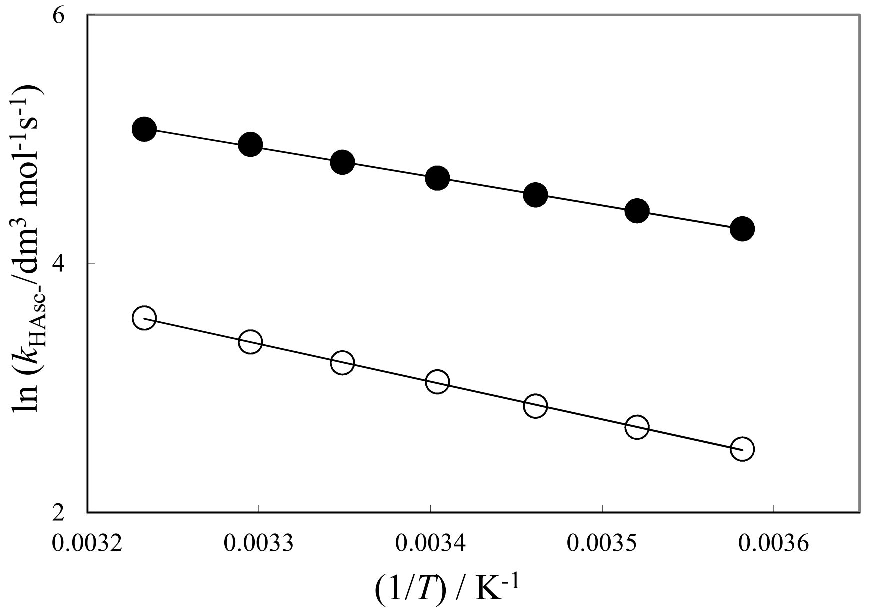

Figure S8. Arrhenius plot for the reaction of ascorbate and hexacyanoferrate(III) ion in presence of 0.01 M tetraethylammonium chloride (TEACl) in H_2O (●), r = 0.999 and D_2O (○), r = 0.999. Data from Table S8.

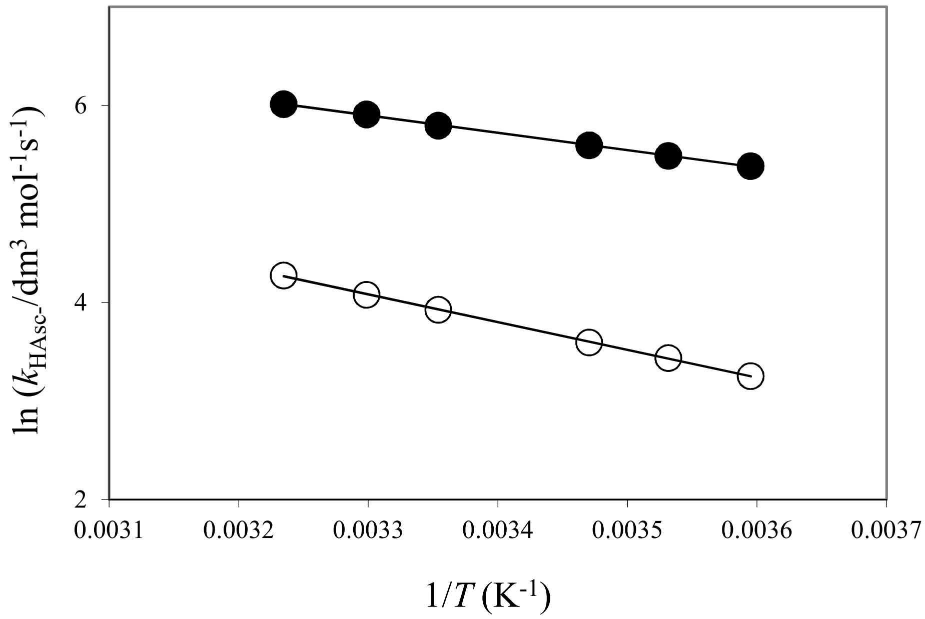

Figure S9. Arrhenius plot for the reaction of ascorbate and hexacyanoferrate(III) ion in presence of 0.1 M benzyltrimethylammonium chloride (BTMACl) in H_2O (●), r = 0.999 and D_2O (○), r = 0.999. Data from Table S9.

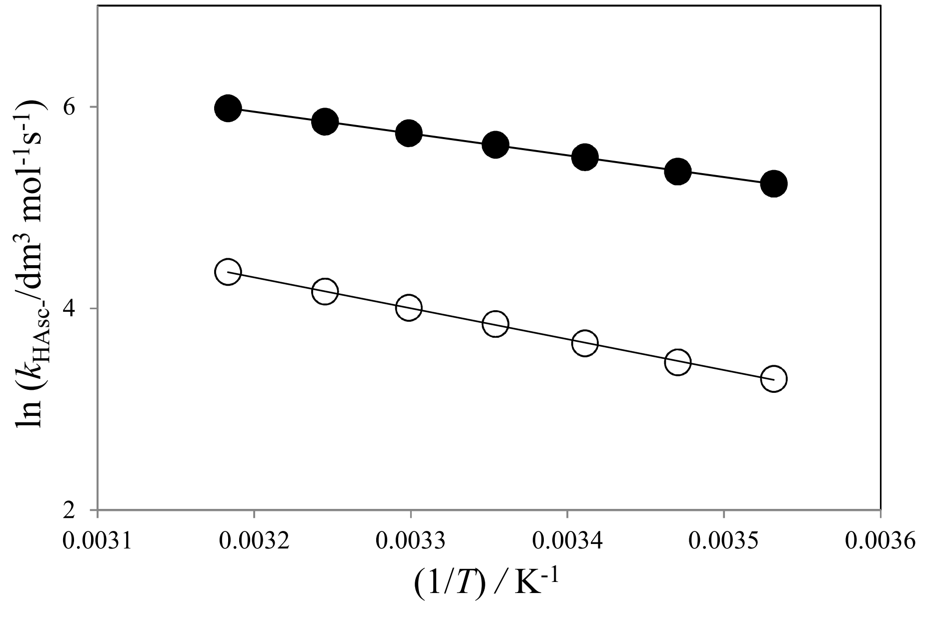

Figure S10. Arrhenius plot for the reaction of ascorbate and hexacyanoferrate(III) ion in presence of 0.1 M acetylcholine chloride (AChCl) in H_2O (●), r = 0.999 and D_2O (○), r = 0.999. Data from Table S10.

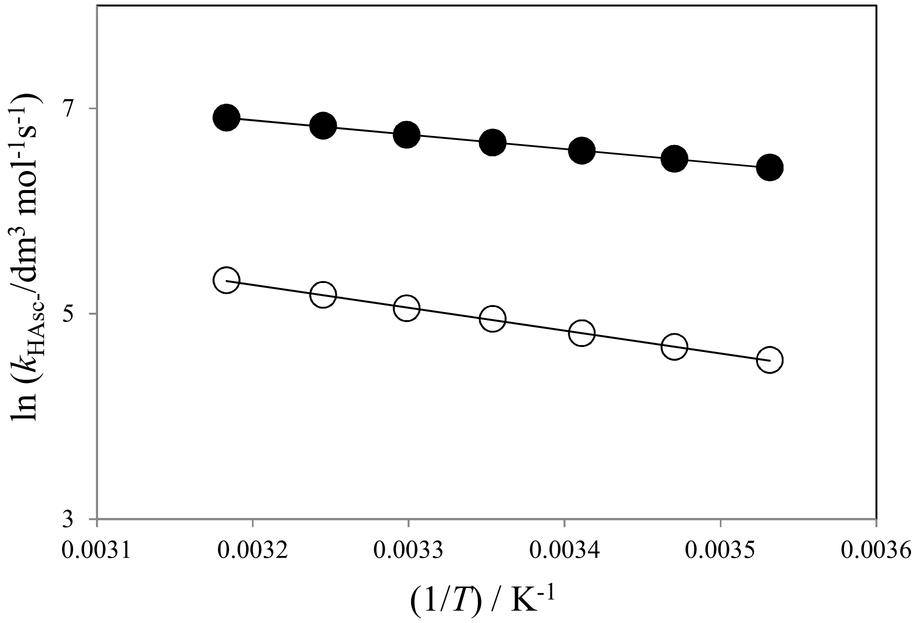

Figure S11. Arrhenius plot for the reaction of ascorbate and hexacyanoferrate(III) ion in presence of 0.006 M paraquat dichloride hydrate ($PQCl_2$) in H_2O (●), r = 0.999 and D_2O (○), r = 0.999. Data from Table S11.

Table S12. Arrhenius Parameters for the Reaction of Ascorbate and Hexacyanoferrate(III) Ion in Water in Presence of Organic Cosolvent or Quaternary Ammonium Ions. Calculated from Plots in Figures S1-S11.

		E_a/kJ mol^{-1}	A_H/A_D
1,4-dioxane : water = 0.05 : 0.95 (v/v)	H_2O	22.9 (0.1)	0.39 (0.03)
	D_2O	29.4 (0.2)	
1,4-dioxane : water = 0.1 : 0.9 (v/v)	H_2O	24.9 (0.4)	0.21 (0.05)
	D_2O	33.3 (0.5)	
MeCN : water = 0.1 : 0.9 (v/v)	H_2O	22.6 (0.1)	0.21 (0.02)
	D_2O	31.1 (0.2)	
EtOH : water = 0.1 : 0.9 (v/v)	H_2O	21.7 (0.2)	0.43 (0.05)

	D_2O	28.1 (0.2)	
c(TMACl) = 0.005 M	H_2O	18.9 (0.1)	0.44 (0.03)
	D_2O	25.1 (0.1)	
c(TPACl) = 0.005 M	H_2O	21.9 (0.0)	0.41 (0.04)
	D_2O	28.3 (0.3)	
c(TBACl) = 0.005 M	H_2O	22.7 (0.2)	0.48 (0.07)
	D_2O	28.7 (0.3)	
c(TEACl) = 0.01 M	H_2O	19.3 (0.2)	0.48 (0.06)
	D_2O	25.2 (0.2)	
c(BTMACl) = 0.1 M	H_2O	14.7 (0.2)	0.19 (0.02)
	D_2O	23.4 (0.2)	
c(AChCl) = 0.1 M	H_2O	18.0 (0.1)	0.29 (0.03)
	D_2O	25.5 (0.3)	
c($PQCl_2$) = 0.006 M	H_2O	11.6 (0.1)	0.35 (0.03)
	D_2O	18.5 (0.2)	

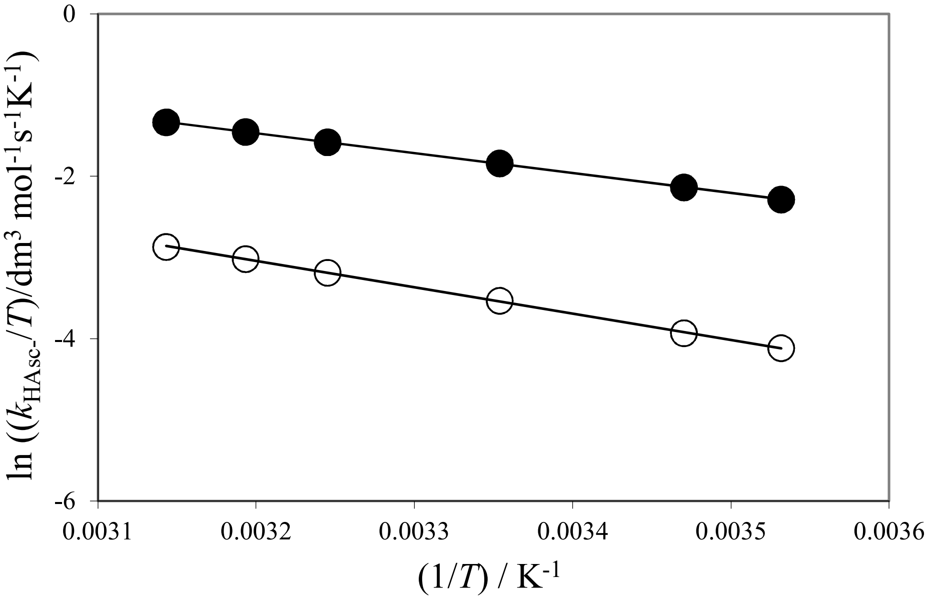

Figure S1A. Eyring plot for the reaction of ascorbate and hexacyanoferrate(III) ion in 1,4-dioxane-H_2O solvent mixture (0.05 : 0.95 v/v) (•), r = 0.999 and 1,4-dioxane-D_2O

solvent mixture (0.05 : 0.95 v/v) (○), r = 0.999, at ionic strength I = 0.0023. Data from Table S1.

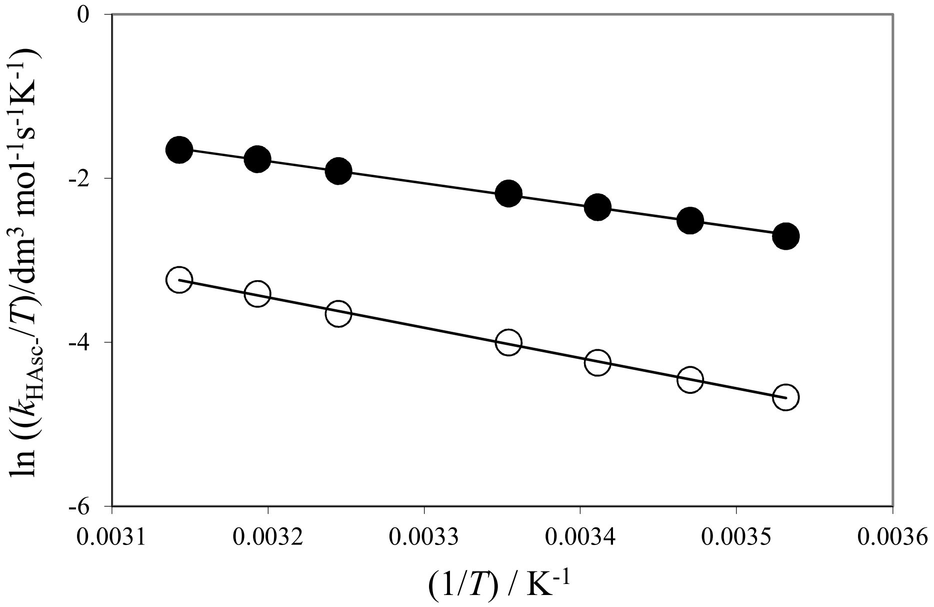

Figure S2A. Eyring plot for the reaction of ascorbate and hexacyanoferrate(III) ion in 1,4-dioxane-H_2O solvent mixture (0.1 : 0.9 v/v) (●), r = 0.999 and 1,4-dioxane-D_2O solvent mixture (0.1 : 0.9 v/v) (○), r = 0.999, at ionic strength I = 0.0023. Data from Table S2.

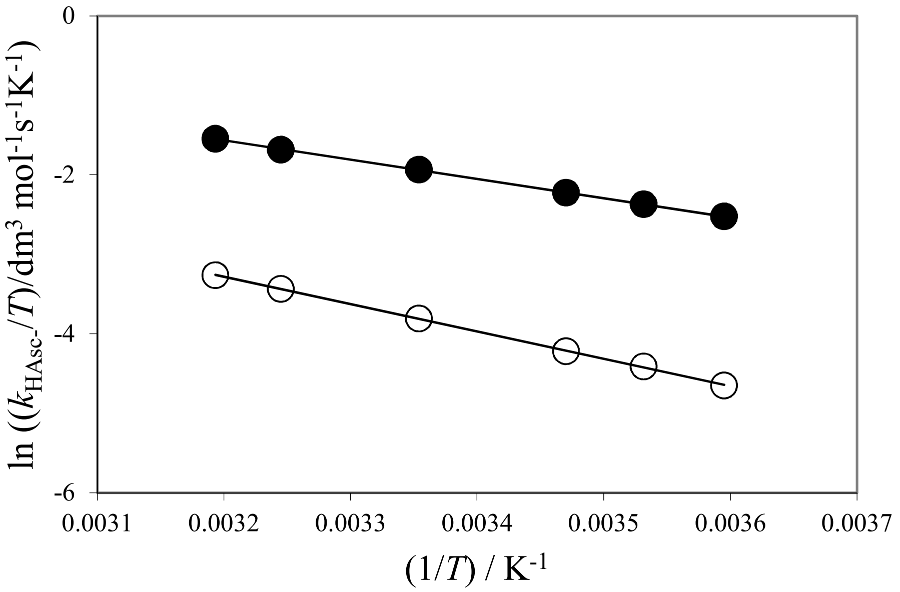

Figure S3A. Eyring plot for the reaction of ascorbate and hexacyanoferrate(III) ion in MeCN-H_2O solvent mixture (0.1 : 0.9 v/v) (●), r = 0.999 and MeCN-D_2O solvent mixture (0.1 : 0.9 v/v) (○), r = 0.999, at ionic strength I = 0.0023. Data from Table S3.

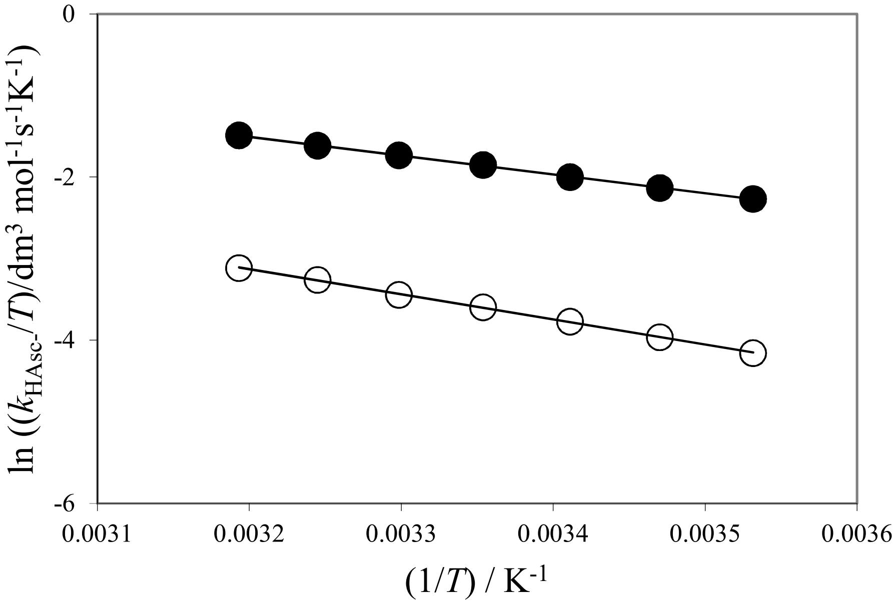

Figure S4A. Eyring plot for the reaction of ascorbate and hexacyanoferrate(III) ion in EtOH-H_2O solvent mixture (0.1 : 0.9 v/v) (●), r = 0.999 and EtOD-D_2O solvent mixture (0.1 : 0.9 v/v) (○), r = 0.999, at ionic strength I = 0.0023. Data from Table S4.

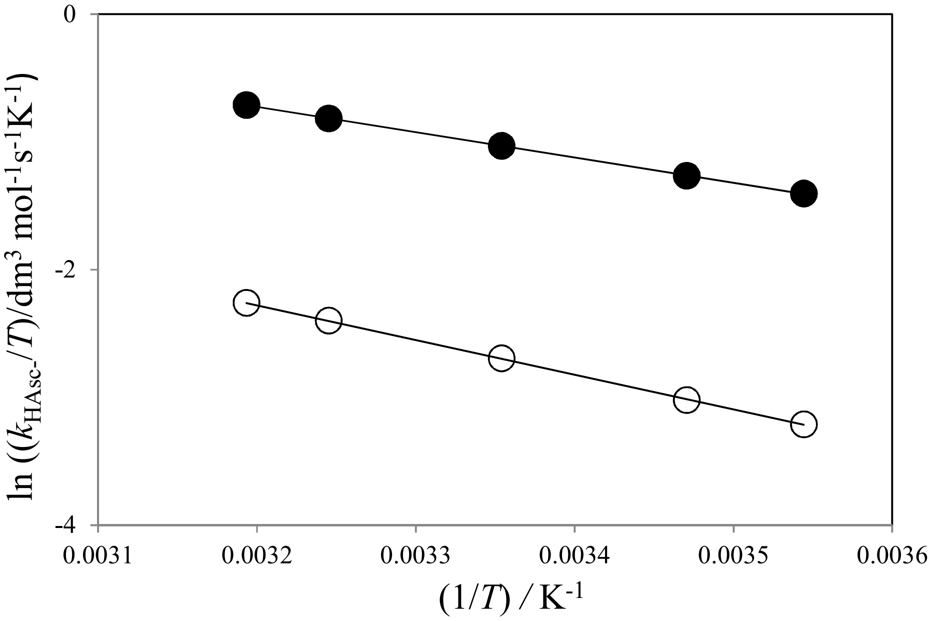

Figure S5A. Eyring plot for the reaction of ascorbate and hexacyanoferrate(III) ion in presence of 0.005 M tetramethylammonium chloride (TMACl) in H_2O (●), r = 0.999 and D_2O (○), r = 0.999. Data from Table S5.

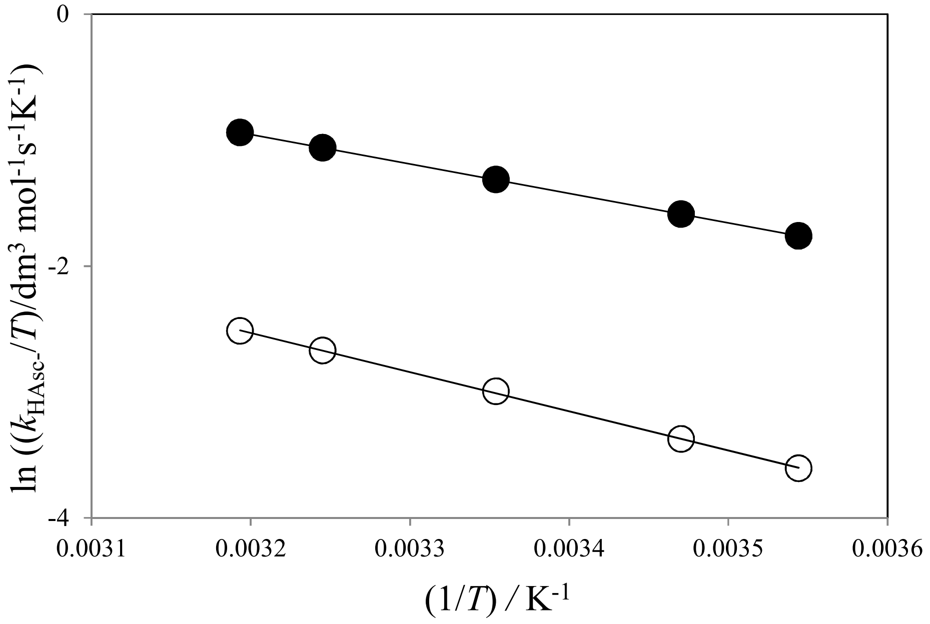

Figure S6A. Eyring plot for the reaction of ascorbate and hexacyanoferrate(III) ion in presence of 0.005 M tetrapropylammonium chloride (TPACl) in H_2O (●), r = 0.999 and D_2O (○), r = 0.999. Data from Table S6.

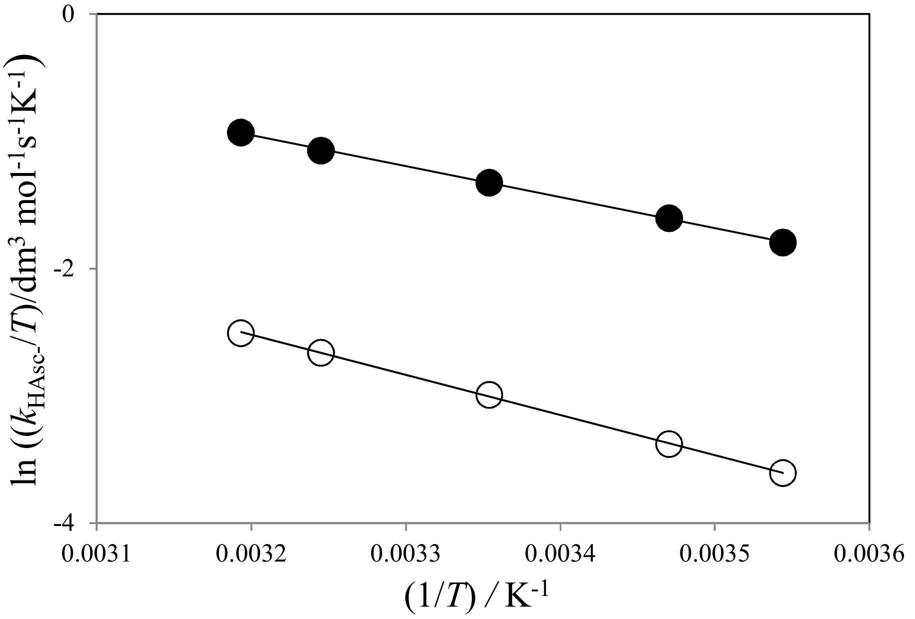

Figure S7A. Eyring plot for the reaction of ascorbate and hexacyanoferrate(III) ion in presence of 0.005 M tetrabutylammonium chloride (TBACl) in H_2O (●), r = 0.999 and D_2O (○), r = 0.999. Data from Table S7.

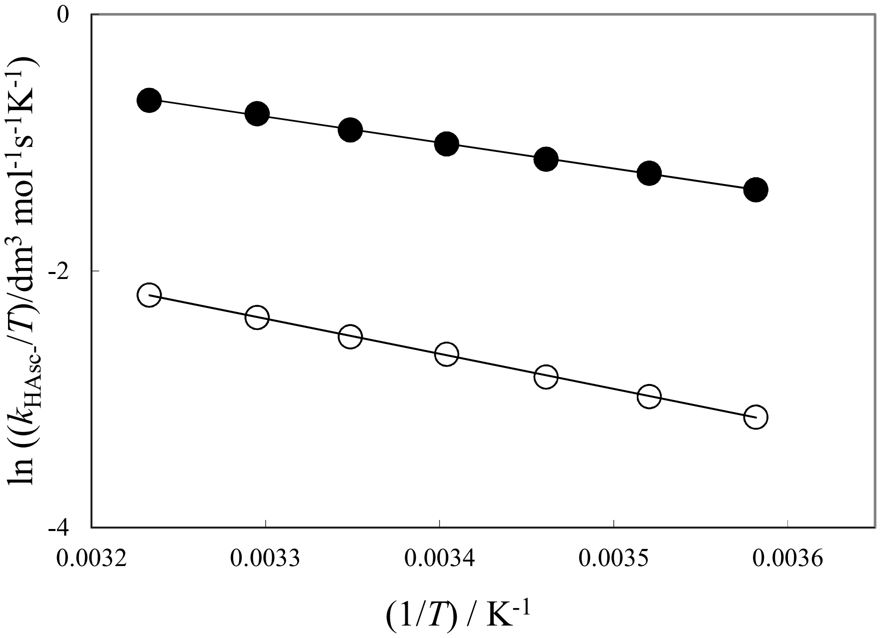

Figure S8A. Eyring plot for the reaction of ascorbate and hexacyanoferrate(III) ion in presence of 0.01 M tetraethylammonium chloride (TEACl) in H_2O (●), r = 0.999 and D_2O (○), r = 0.999. Data from Table S8.

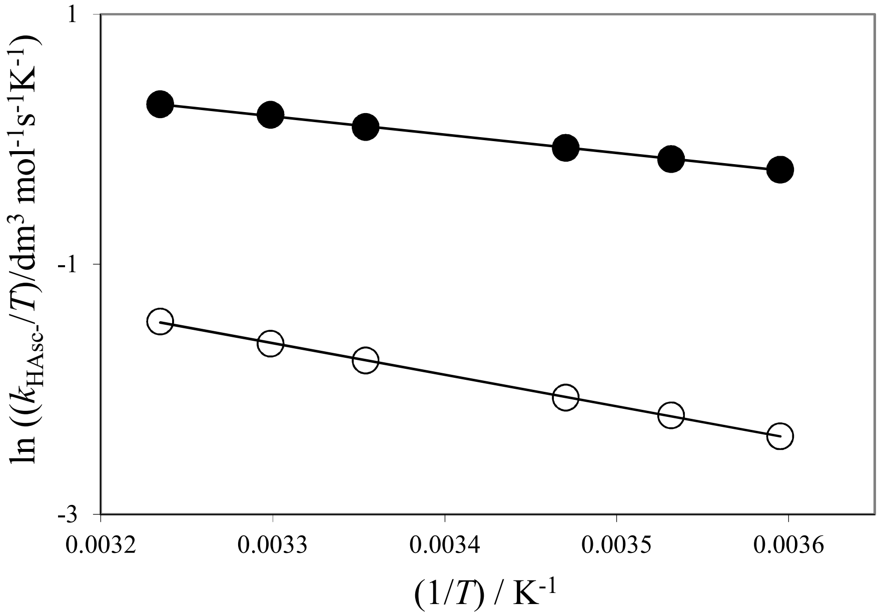

Figure S9A. Eyring plot for the reaction of ascorbate and hexacyanoferrate(III) ion in presence of 0.1 M benzyltrimethylammonium chloride (BTMACl) in H_2O (●), r = 0.999 and D_2O (○), r = 0.999. Data from Table S9.

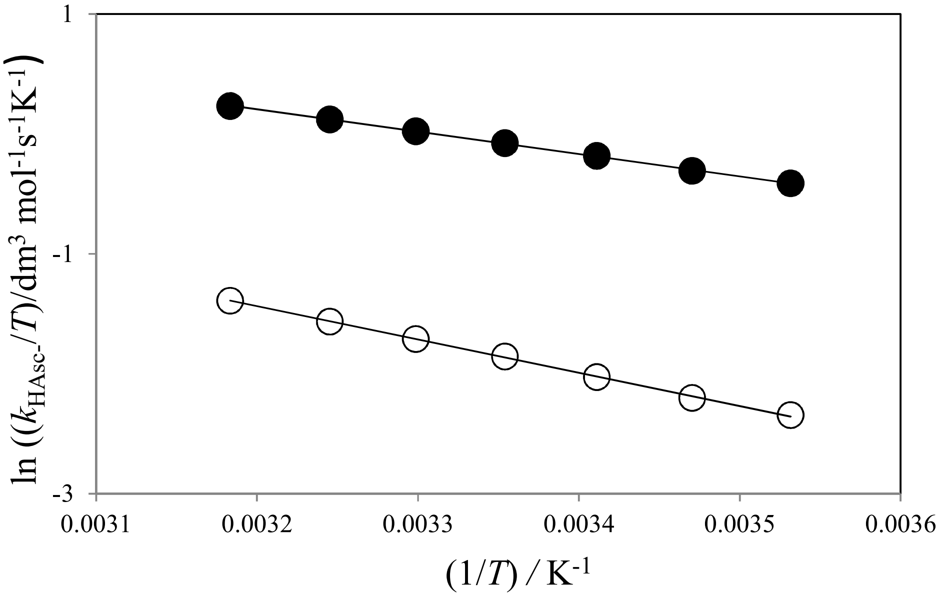

Figure S10A. Eyring plot for the reaction of ascorbate and hexacyanoferrate(III) ion in presence of 0.1 M acetylcholine chloride (AChCl) in H_2O (•), r = 0.999 and D_2O (○), r = 0.999. Data from Table S10.

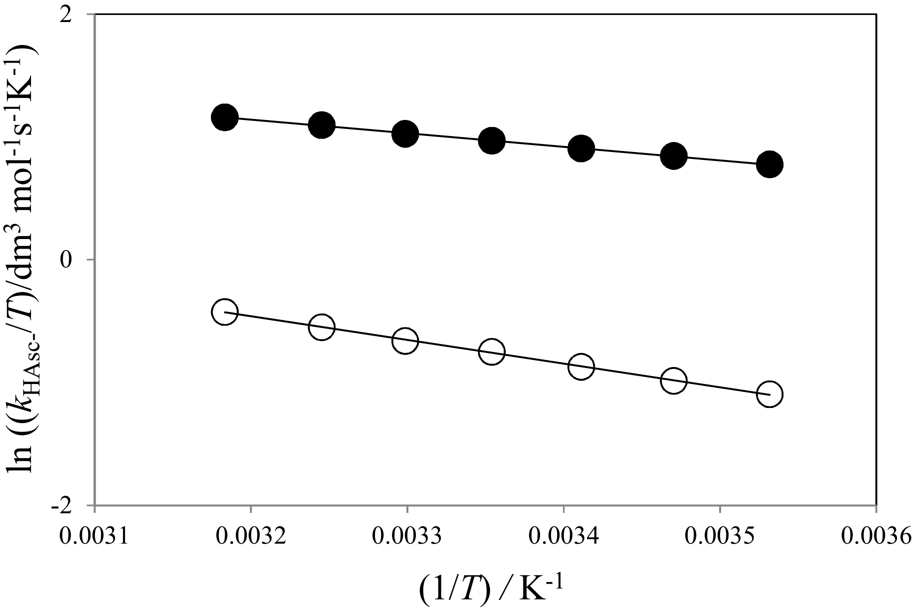

Figure S11A. Eyring plot for the reaction of ascorbate and hexacyanoferrate(III) ion in presence of 0.006 M paraquat dichloride hydrate ($PQCl_2$) in H_2O (•), r = 0.999 and D_2O (○), r = 0.999. Data from Table S11.

Table S13. Eyring Parameters for the Reaction of Ascorbate and Hexacyanoferrate(III) Ion in Water in Presence of Organic Cosolvent or Quaternary Ammonium Ions. Calculated from Plots in Figures S1A-S11A.

		$\Delta H^‡$/ kJ mol^{-1}	$\Delta S^‡$/ J K^{-1} mol^{-1}	$\Delta G^{‡a}$/ kJ mol^{-1}	$\Delta\Delta H^‡$(D,H) /kJ mol^{-1}	$\Delta\Delta S^‡$(D,H) /J K^{-1} mol^{-1}
1,4-dioxane : water = 0.05 : 0.95 (v/v)	H_2O	20.4 (0.1)	-144.4 (0.3)	63.5 (0.1)	6.5 (0.2)	7.8 (0.8)
	D_2O	26.9 (0.2)	-136.6 (0.7)	67.7 (0.3)		
1,4-dioxane : water = 0.1 : 0.9 (v/v)	H_2O	22.4 (0.4)	-140.7 (1.2)	64.4 (0.5)	8.4 (0.6)	12.9 (2.1)
	D_2O	30.8 (0.5)	-127.8 (1.7)	68.9 (0.7)		
MeCN : water = 0.1 : 0.9 (v/v)	H_2O	20.1 (0.1)	-146.2 (0.4)	63.7 (0.2)	8.6 (0.2)	13.1 (0.8)
	D_2O	28.7 (0.2)	-133.1 (0.7)	68.3 (0.3)		
EtOH : water = 0.1 : 0.9 (v/v)	H_2O	19.2 (0.2)	-148.6 (0.6)	63.5 (0.3)	6.4 (0.3)	7.0 (1.0)
	D_2O	25.6 (0.2)	-141.6 (0.8)	67.8 (0.3)		
c(TMACl) = 0.005 M	H_2O	16.5 (0.1)	-150.8 (0.2)	61.5 (0.1)	6.2 (0.4)	6.8 (1.1)
	D_2O	22.7 (0.1)	-144.0 (0.5)	65.6 (0.2)		
c(TPACl) = 0.005 M	H_2O	19.4 (0.0)	-143.3 (0.1)	62.1 (0.0)	6.5 (0.4)	7.3 (1.1)
	D_2O	25.9 (0.3)	-136.0 (0.8)	66.4 (0.4)		
c(TBACl) = 0.005 M	H_2O	20.2 (0.2)	-140.8 (0.7)	62.2 (0.3)	6.0 (0.4)	6.2 (1.1)
	D_2O	26.2 (0.3)	-134.6 (0.9)	66.3 (0.4)		
c(TEACl) = 0.01 M	H_2O	16.8 (0.4)	-149.4 (0.7)	61.4 (0.3)	5.9 (0.3)	6.0 (1.0)
	D_2O	22.7 (0.2)	-143.4 (0.7)	65.5 (0.3)		
c(BTMACl) = 0.1 M	H_2O	12.3 (0.2)	-155.7 (0.6)	58.6 (0.2)	8.7 (0.2)	13.8 (0.8)
	D_2O	21.0 (0.2)	-141.9 (0.6)	63.3 (0.3)		
c(AChCl) = 0.1 M	H_2O	15.5 (0.1)	-146.3 (0.5)	59.1 (0.2)	7.5 (0.3)	10.4 (1.0)
	D_2O	23.0 (0.3)	-135.9 (0.9)	63.5 (0.4)		
c($PQCl_2$) = 0.006 M	H_2O	9.2 (0.1)	-158.8 (0.4)	56.5 (0.2)	6.8 (0.2)	8.7 (0.8)
	D_2O	16.0 (0.2)	-150.1 (0.6)	60.8 (0.3)		

[a] 298.15 K

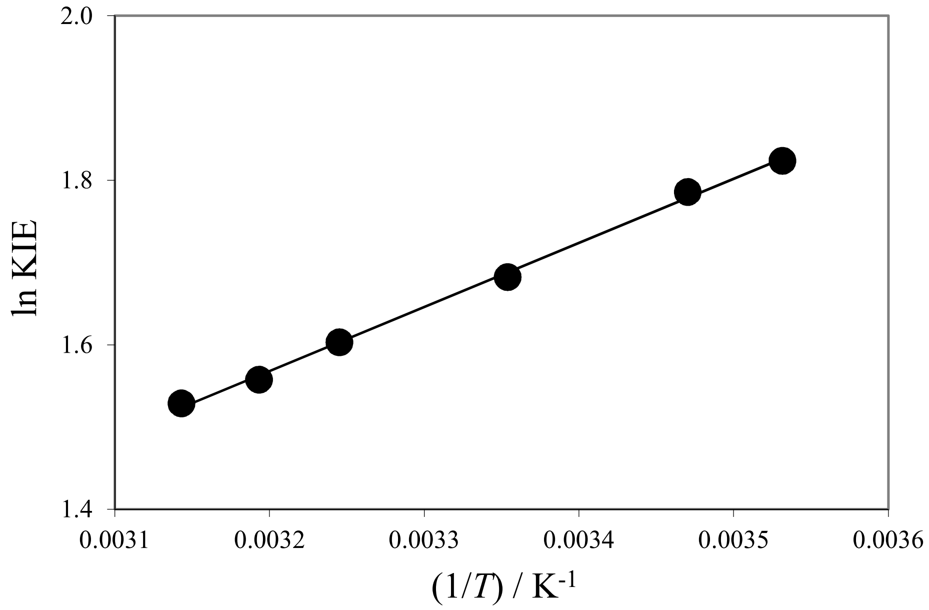

Figure S1B. Dependence of lnKIE on $1/T$ in the reaction of ascorbate with hexacyanoferrate(III) ion in 1,4-dioxane-water solvent mixture (0.05 : 0.95 v/v) (●). Data from Table S1.

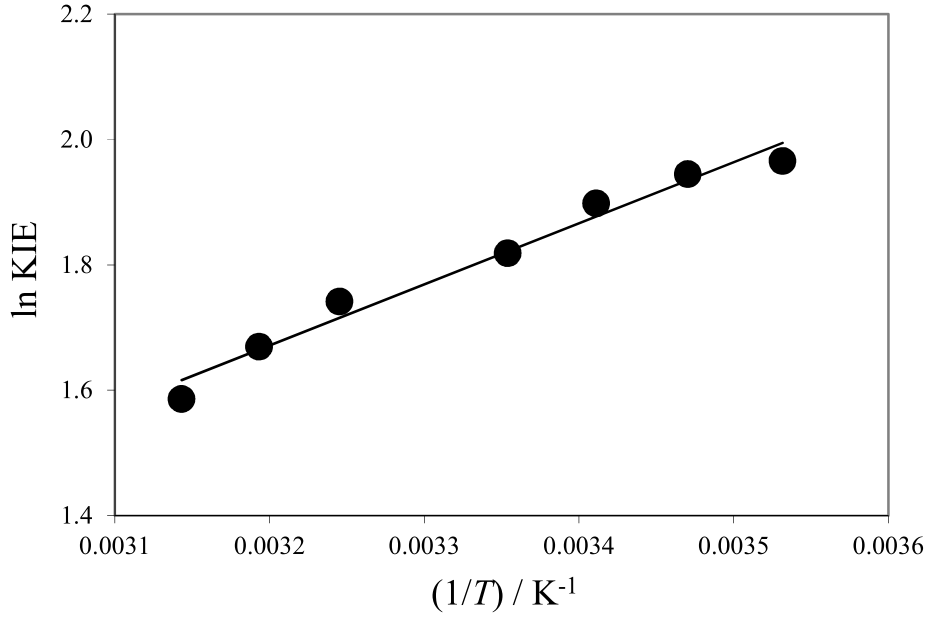

Figure S2B. Dependence of lnKIE on $1/T$ in the reaction of ascorbate with hexacyanoferrate(III) ion in 1,4-dioxane-water solvent mixture (0.1 : 0.9 v/v) (●). Data from Table S2.

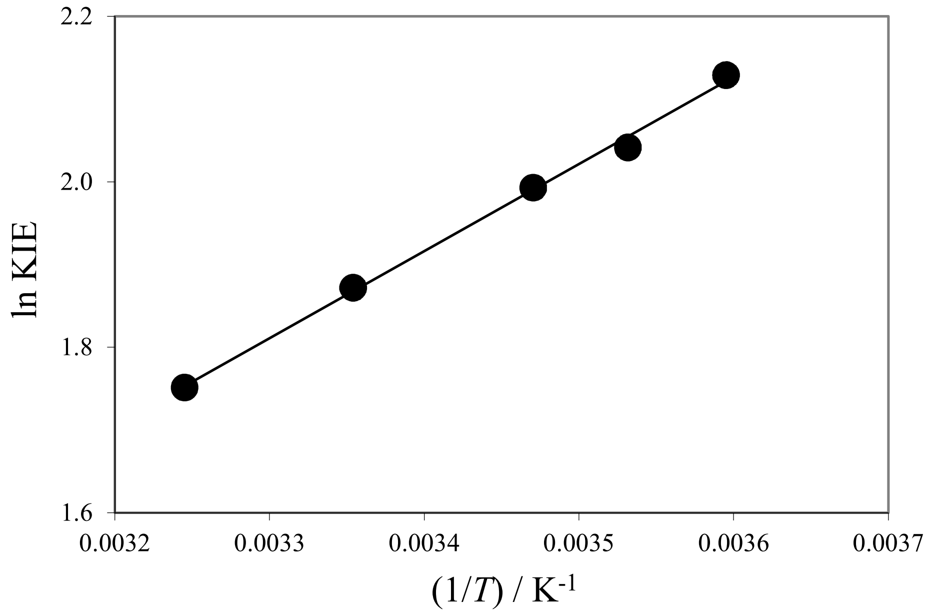

Figure S3B. Dependence of lnKIE on 1/T in the reaction of ascorbate with hexacyanoferrate(III) ion in MeCN-water solvent mixture (0.1 : 0.9 v/v) (●). Data from Table S3.

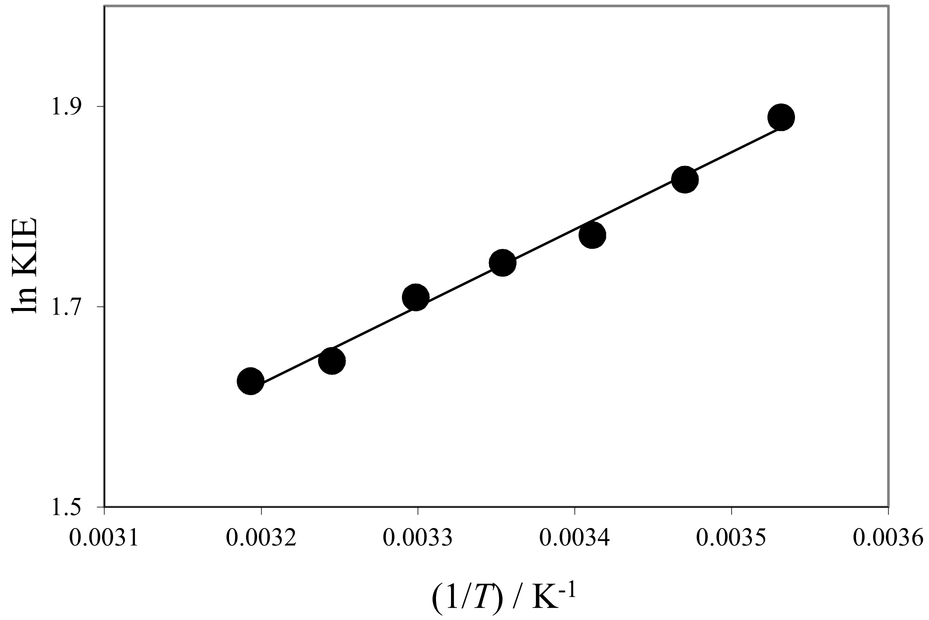

Figure S4B. Dependence of lnKIE on 1/T in the reaction of ascorbate with hexacyanoferrate(III) ion in ethanol-water solvent mixture (0.1 : 0.9 v/v) (●). Data from Table S4.

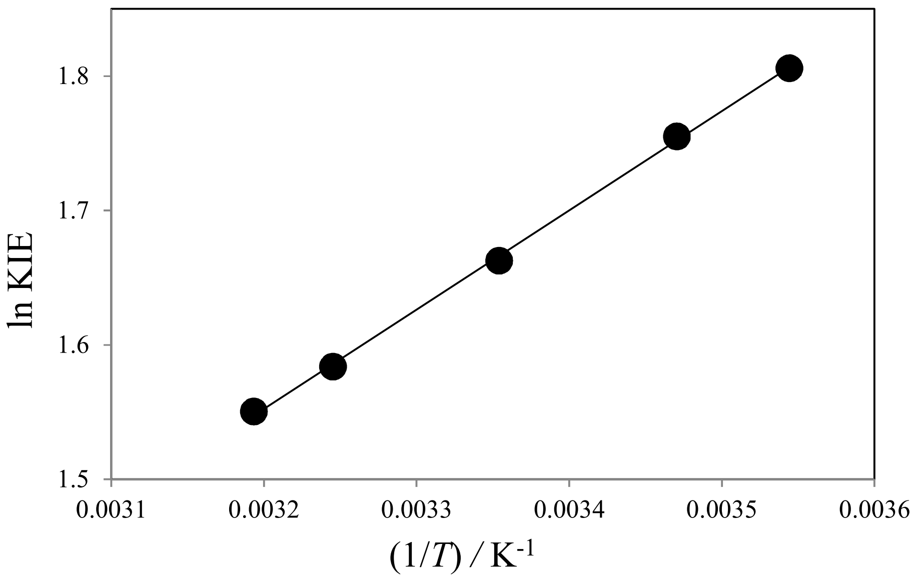

Figure S5B. Dependence of lnKIE on 1/*T* in the reaction of ascorbate with hexacyanoferrate(III) ion in presence of 0.005 M tetramethylammonium chloride (TMACl) in water. Data from Table S5.

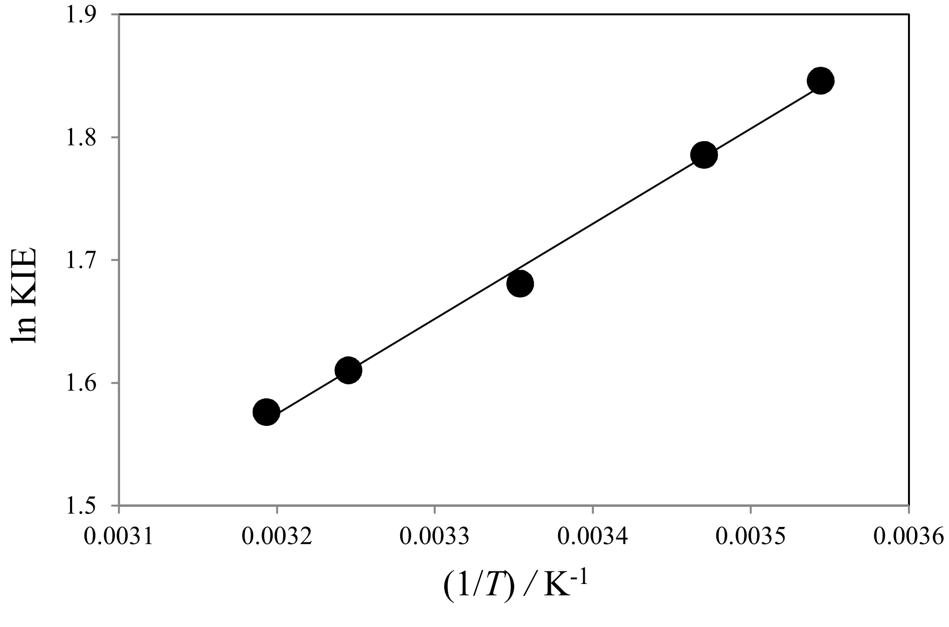

Figure S6B. Dependence of lnKIE on 1/*T* in the reaction of ascorbate with hexacyanoferrate(III) ion in presence of 0.005 M tetrapropylammonium chloride (TPACl) in water. Data from Table S6.

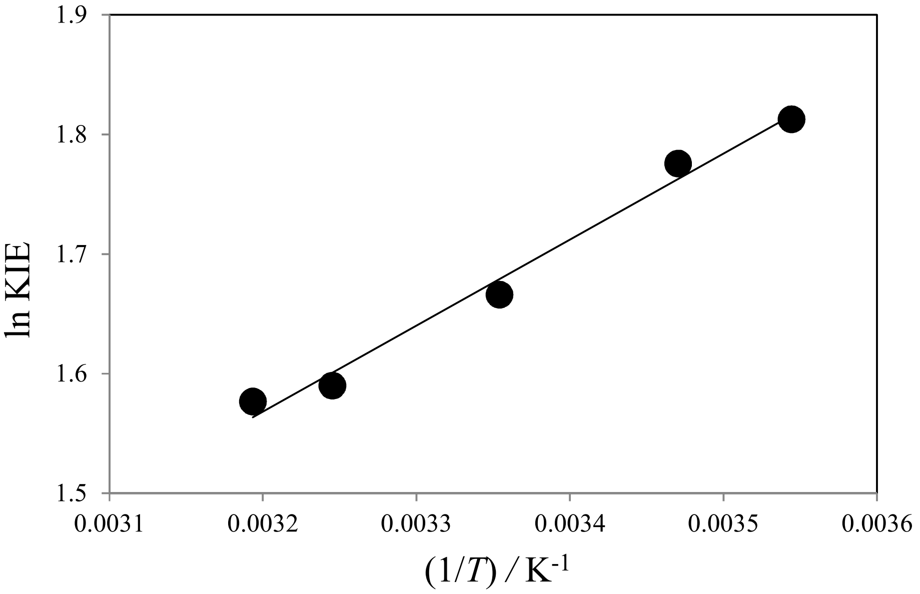

Figure S7B. Dependence of lnKIE on 1/*T* in the reaction of ascorbate with hexacyanoferrate(III) ion in presence of 0.005 M tetrabutylammonium chloride (TBACl) in water. Data from Table S7.

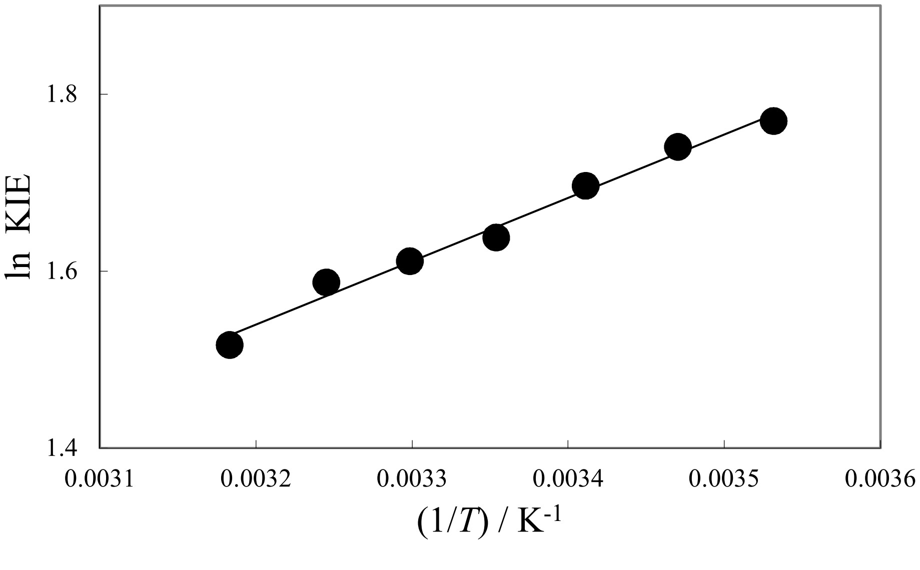

Figure S8B. Dependence of lnKIE on 1/*T* in the reaction of ascorbate with hexacyanoferrate(III) ion in presence of 0.01 M tetraethylammonium chloride (TEACl) in water. Data from Table S8.

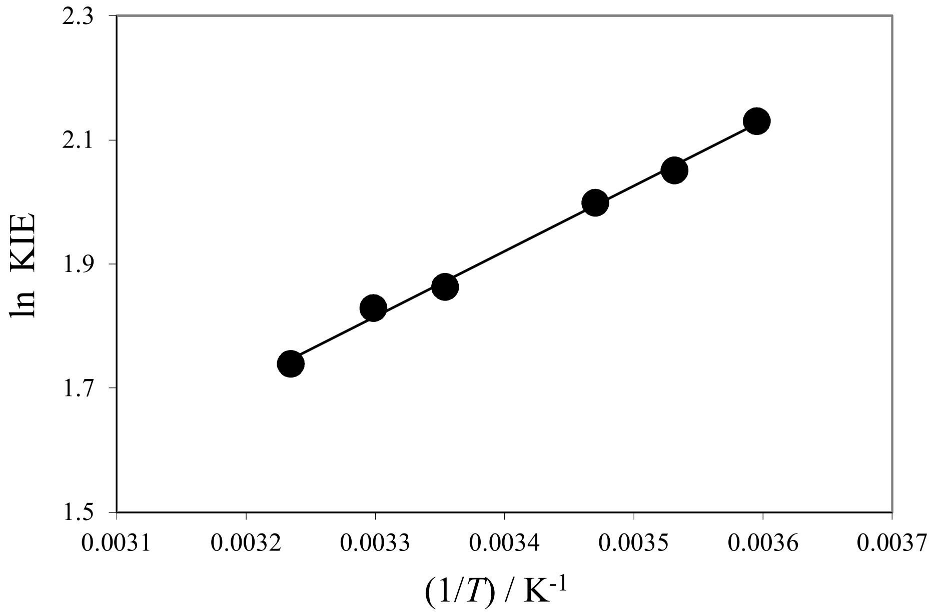

Figure S9B. Dependence of lnKIE on 1/*T* in the reaction of ascorbate with hexacyanoferrate(III) ion in presence of 0.1 M benzyltrimethylammonium chloride (BTMACl) in water. Data from Table S9.

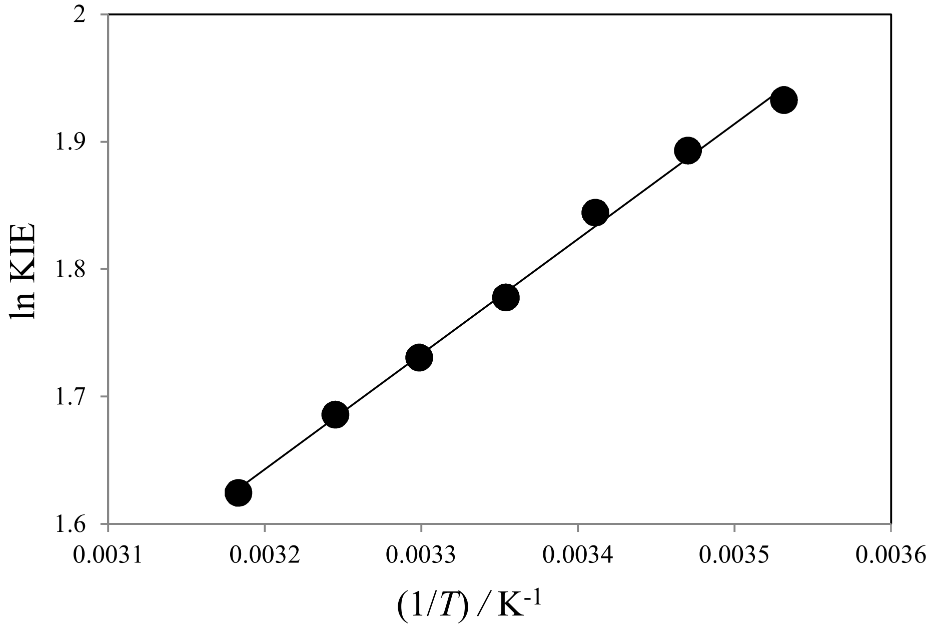

Figure S10B. Dependence of lnKIE on 1/*T* in the reaction of ascorbate with hexacyanoferrate(III) ion in presence of 0.1 M acetylcholine chloride (AChCl) in water. Data from Table S10.

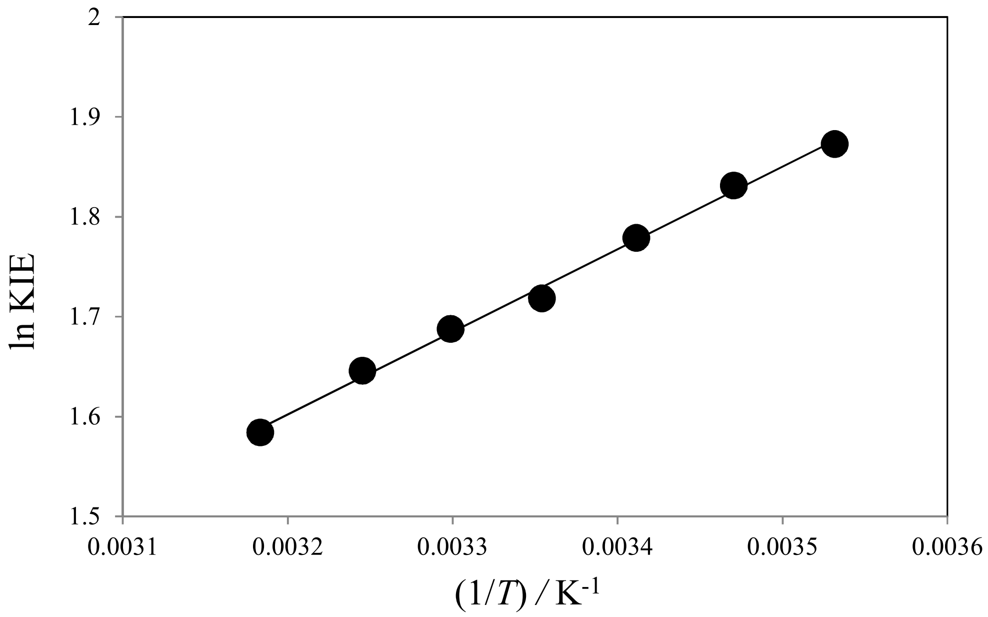

Figure S11B. Dependence of lnKIE on $1/T$ in the reaction of ascorbate with hexacyanoferrate(III) ion in presence of 0.006 M paraquat dichloride hydrate ($PQCl_2$) in water. Data from Table S11.

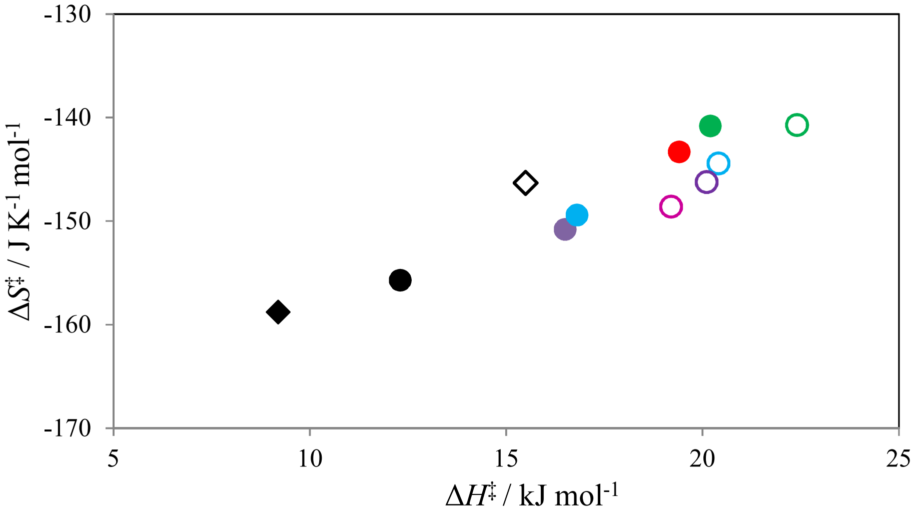

Figure S12. Relationship between $\Delta H^{\ddagger}$ and $\Delta S^{\ddagger}$ for the reaction of ascorbate and hexacyanoferrate(III) ion in H_2O in presence of organic cosolvent or quaternary ammonium ions. Data from Table S13. Key: violet circle, 0.005 M TMACl; blue circle, 0.01 M TEACl; red circle, 0.005 M TPACl; green circle, 0.005 M TBACl; black circle, 0.1 M

BTMACl; hollow black diamond, 0.1 M AChCl; black diamond, 0.006 $PQCl_2$; hollow blue circle, 1,4-dioxane : water = 0.05 : 0.95 v/v; hollow green circle, 1,4-dioxane : water = 0.1 : 0.9 v/v; hollow violet circle, MeCN : water = 0.1 : 0.9 v/v; hollow magenta circle, EtOH : water = 0.1 : 0.9 v/v. Abbreviations as noted in Manuscript.

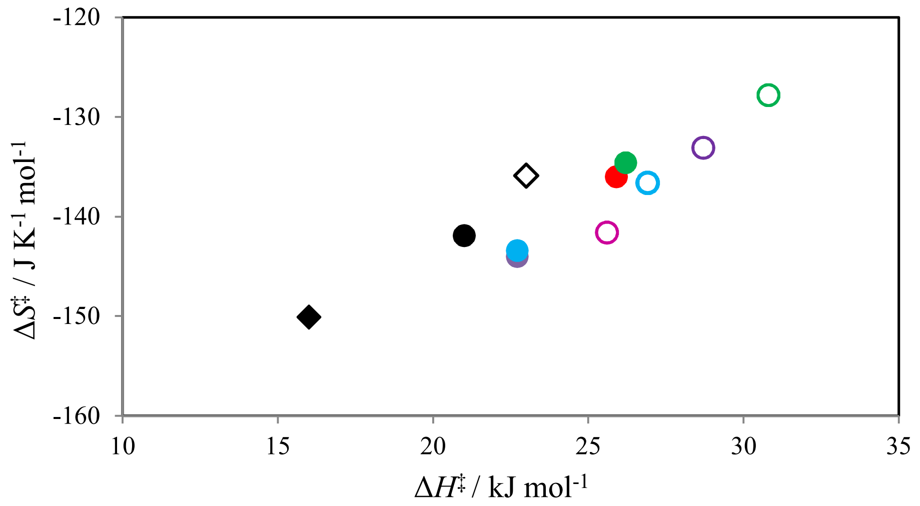

Figure S13. Relationship between $\Delta H^‡$ and $\Delta S^‡$ for the reaction of ascorbate and hexacyanoferrate(III) ion in D_2O in presence of organic cosolvent or quaternary ammonium ions. Data from Table S13. Key: violet circle, 0.005 M TMACl; blue circle, 0.01 M TEACl; red circle, 0.005 M TPACl; green circle, 0.005 M TBACl; black circle, 0.1 M BTMACl; hollow black diamond, 0.1 M AChCl; black diamond, 0.006 $PQCl_2$; hollow blue circle, 1,4-dioxane : water = 0.05 : 0.95 v/v; hollow green circle, 1,4-dioxane : water = 0.1 : 0.9 v/v; hollow violet circle, MeCN : water = 0.1 : 0.9 v/v; hollow magenta circle, EtOH : water = 0.1 : 0.9 v/v. Abbreviations as noted in Manuscript.

4. Thermochemistry

Thermochemical analysis for the reaction of ascorbate and hexacyanoferrate(III) ion in different solvent-water mixtures can be rationalized starting from the thermochemical analysis in water (see Scheme S2 below).* The addition of organic cosolvents leads to increased pK_a values and decreased magnitudes of $E^{o\prime}(Fe(CN)_6^{3-}/Fe(CN)_6^{4-})$ (see Figures S12 and S13; $E^{o\prime}(Fe(CN)_6^{3-}/Fe(CN)_6^{4-}$ correlates linearly with expanded Debye-Hückel equation) and consequently to the increased driving force relatively to the situation in the water solution.

Thermochemistry of L-ascorbate in water:

$pK_a(HAsc^-) = 11.34$ (at $I = 0.1$)

$pK_a(HAsc\bullet) = -0.45$

$E^{o\prime}(HAsc\bullet/HAsc^-) = 0.720$ mV

$E^{o\prime}(Asc\bullet^-/Asc^{2-}) = 0.019$ mV

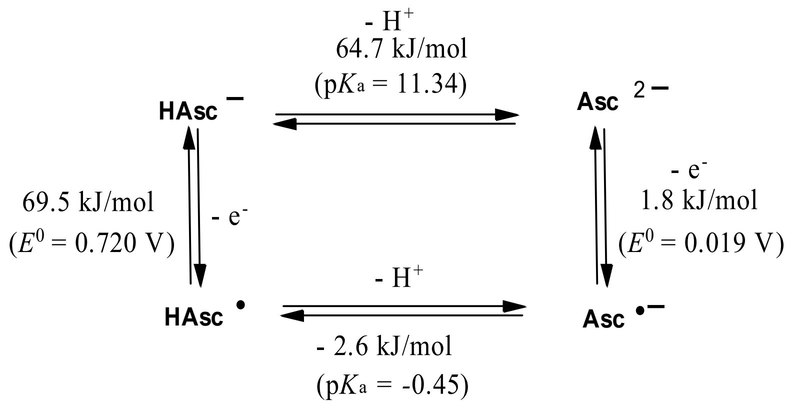

Scheme S2. Thermochemistry of L-ascorbate in water

Thermochemical analysis for the reaction of ascorbate and hexacyanoferrate(III) ion in water:

ET/PT

$Fe(CN)_6^{3-} + HAsc^- \rightarrow Fe(CN)_6^{4-} + HAsc^\bullet$

Free energy barrier for the initial ET in the sequential ET/PT calculated taking into account $E^{o\prime}(Fe(CN)_6^{3-}/Fe(CN)_6^{4-})$ = 0.411 V (I = 0.102) and $E^{o\prime}(HAsc^\bullet/HAsc^-)$ = 0.720 V should be greater or (utmost) equal 31.0 kJ/mol, the calculated driving force for the above process. (The observed activation barrier in the reaction is 64.2 kJ/mol.)

PT/ET

$Fe(CN)_6^{3-} + HAsc^- \rightarrow Fe(CN)_6^{3-},Asc^{2-} + H^+$

Driving force for the initial PT in the sequential PT/ET can be calculated as the sum of:

(1) $HAsc^- \rightarrow Asc^{2-} + H^+$ (dissociation of ascorbate monoanion, pK_a 11.3) and

(2) $Fe(CN)_6^{3-} + Asc^{2-} \rightarrow Fe(CN)_6^{3-},Asc^{2-}$ (formation of encounter pair $Fe(CN)_6^{3-},Asc^{2-}$, K ~ 0.1), what gives 70.2 kJ/mol.

PCET

$Fe(CN)_6^{3-} + HAsc^- \rightarrow Fe(CN)_6^{4-} + Asc^{\bullet-} + H^+$

The minimum free energy barrier for the concerted PCET could be (in principle) equal to the driving force for the reaction and can be calculated taking into account $E^{o\prime}(Fe(CN)_6^{3-}/Fe(CN)_6^{4-})$ = 0.411 V (I = 0.102) and $E^o(Asc^{\bullet-},H^+/HAsc^-)$ = 0.693 V,

$E^o(Asc^{\bullet-},H^+/HAsc^-) = \Delta G^o(Asc^{\bullet-},H^+/HAsc^-)/F$

$\Delta G^o(Asc^{\bullet-},H^+/HAsc^-) = FE^o(HAsc^\bullet/HAsc^-)$[4] $+ 2.303RTpK_a(HAsc^\bullet)$

and would be 28.5 kJ/mol. The observed activation barrier in the reaction is 64.2 kJ/mol.

Note: $E^{o\prime}(Fe(CN)_6^{3-}/Fe(CN)_6^{4-})$ decrease with the addition of organic cosolvent; the addition of salt leads to an increase of the $E^{o\prime}$ (the increase depended on $I^{1/2}/(I^{1/2}+1)$ linearly, see Figures S12 and S13.)

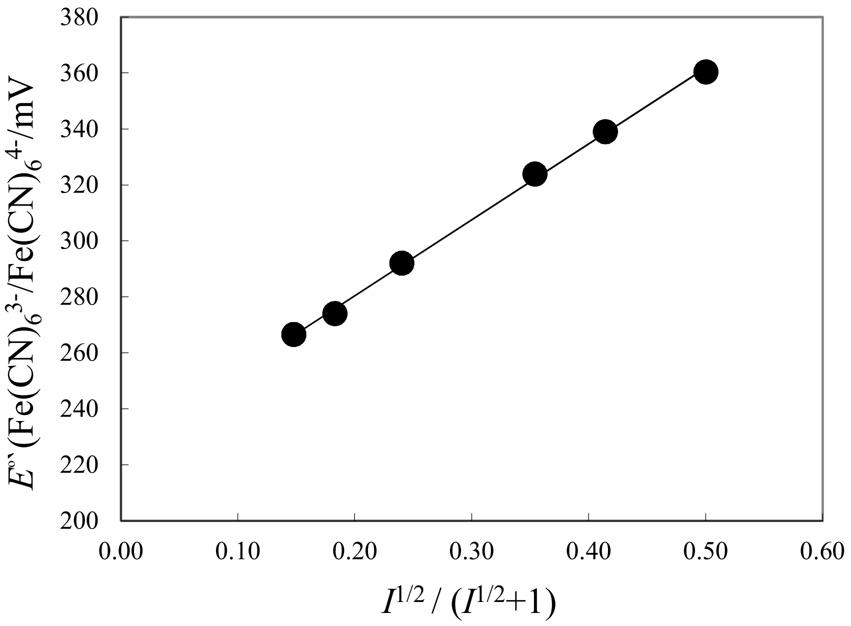

Figure S12. Dependence of $E^{o\prime}(Fe(CN)_6^{3-}/Fe(CN)_6^{4-})$ on $(\sqrt{I}/(\sqrt{I}+1)$, in dioxane – water solvent mixture (1:1 v/v), at 25.0°C (r =0.999). The ionic strength was varied by adding KCl.

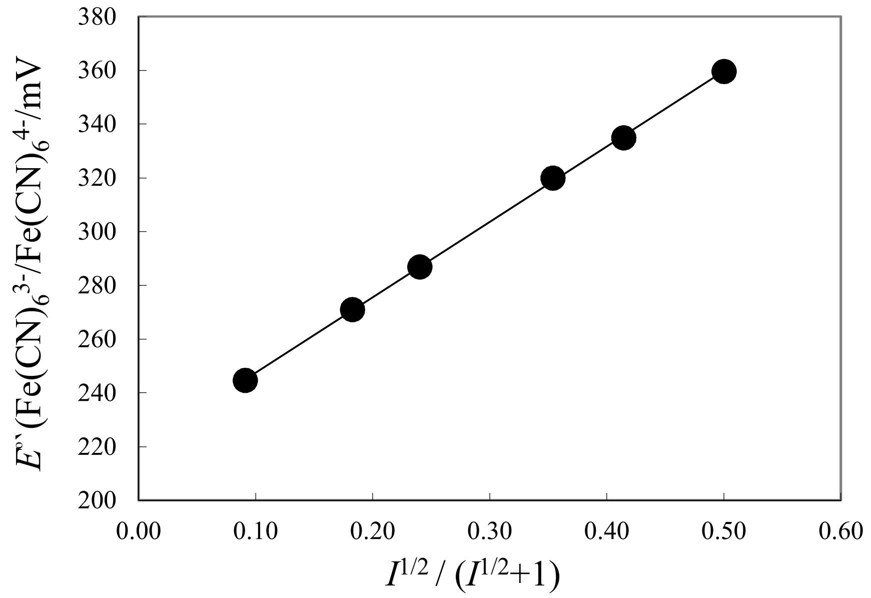

Figure S13. Dependence of $E^{o\prime}(Fe(CN)_6^{3-}/Fe(CN)_6^{4-})$ on $(\sqrt{I}/(\sqrt{I}+1))$, in MeCN – water solvent mixture (1:1 v/v), at 25.0°C (r =0.999). The ionic strength was varied by adding KCl.

Cyclic Voltammetry. Cyclic voltammograms were collected using a Princeton Applied Research Potentiostat/Galvanostat Model 273A. Electrochemical measurements were performed in a N_2 filled cell taking 0.0005 M potassium hexacyanoferrate(III) and varying the concentration of KCl as supporting electrolyte, in dioxane-water solvent mixture (1:1 v/v), at 25°C. The three-electrode arrangement was used: working electrode was the platinum electrode; reference electrode was the saturated calomel electrode; and as counter electrode platinum electrode was used. Scans were taken at 20, 50, 80 and 100 mVs^{-1}. All the potentials are referred to the standard hydrogen electrode (SHE).

* references 19 and 20 in manuscript

5. Ion Pairing in the Presence of Quaternary Ions

The slope of the straight line of log (k/k_o) vs. $I^{1/2}$, (the square root of ionic strength, Brønsted-Debye-Hückel equation) for the reaction in water in the presence of Na^+ or K^+ is 3.0, revealing the interaction between $[Fe(CN)_6]^{3-}$ and $HAsc^-$ ion.* However, in the presence of quaternary ammonium ions, markedly curved dependences of log (k/k_o) *vs* $I^{1/2}$ were obtained with certain of ions. The observation should be consistent with the increasing formation of ion pairs between the $[Fe(CN)_6]^{3-}$ and the quaternary ions added (an example is presented in Figure S14). Furthermore, in the case of TEACl the deviation from the Brønsted-Debye-Hückel straight line is unnoticeable up to $I^{1/2} \sim 0.1$. In other cases, the changes of rate constants observed are in the range expected when there is an ion pairing (see for example Figure S14.)

The formation of ion pairs however, at least in principle, could lead to a change of KIE in the reaction because of possible influence of the pairing on the donor-acceptor distance (DAD) and/or change of reaction driving force (through $E^{o'}$ of hexacyanoferrate(III) ion). But DAD should decrease in a case of formation of ion pairs between the quaternary ammonium ions and hexacyanoferrate(III) ions (because there should be less electrostatic repulsion between ascorbate and hexacyanoferrate anions). Therefore, expectation would be a decrease of KIE. In contrast, increased KIE-s were observed.

Furthermore, the formation of ion pairs, at least in principle, could lead to a change of KIE-s in the reaction due to change of the driving force of the reaction as ion-pairing (because of the electrostatic interaction between the ions) might influence the $E^{o'}$ of hexacyanoferrate(III) ion. However, these small changes of driving force would not alter KIE-s in the process.**

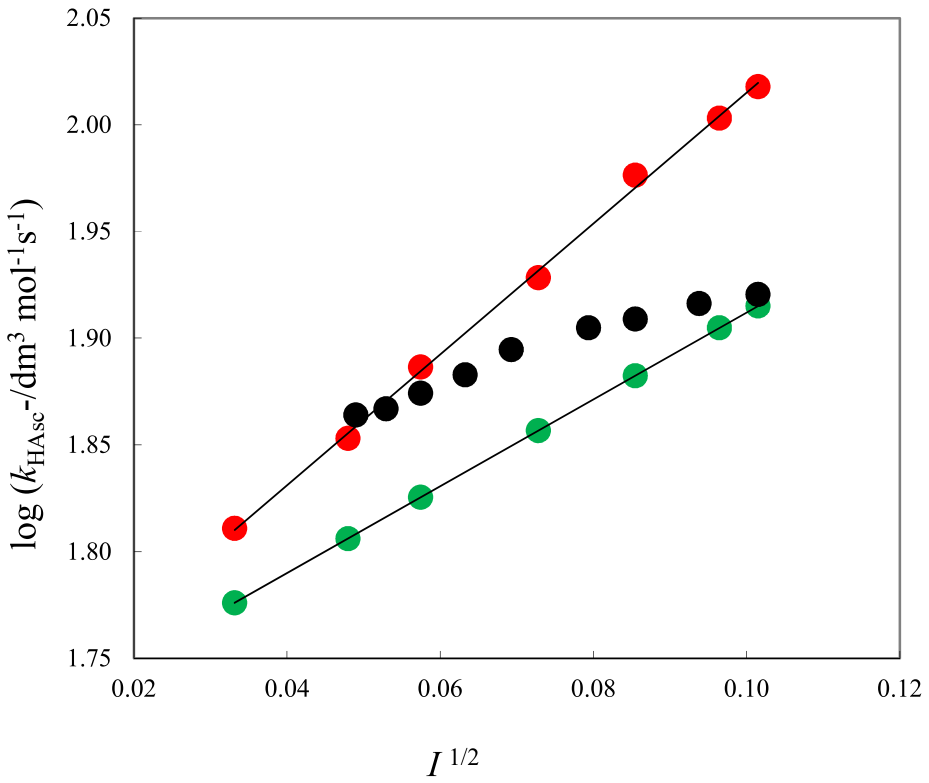

Figure S14. Dependence of log k_{HAsc^-} on square root of ionic strength for the reaction of ascorbate with hexacyanoferrate(III) ion in water, at 25°C. The ionic strength was varied by adding NaCl (●), r = 0.999, y = 3.0658x + 1.7085 (data from Ref. 1); and by adding TBACl (●), data from Table S14. (●), rate constants were calculated taking z_Az_B = 2.0 and log $k_{HAsc^-,0}$ = 1.7085.

Table S14. Rate Constants for the Reaction of Ascorbate with Hexacyanoferrate(III) Ion in Water at Various Concentrations of Tetrabutylammonium Chloride (TBACl), at 25.0°C.

c(TBACl)/M	*I*	k_{HAsc^-}/M^{-1}s^{-1}
0.0001	0.0024	73.1 (0.8)
0.0005	0.0028	73.6 (1.2)
0.001	0.0033	74.9 (0.3)
0.0017	0.0040	76.4 (1.3)
0.0025	0.0048	78.5 (1.2)
0.004	0.0063	80.3 (0.5)
0.005	0.0073	81.1 (1.5)
0.0065	0.0088	82.5 (1.1)
0.008	0.0103	83.3 (1.4)

$5 \cdot 10^{-4}$ M Na-ascorbate, $5 \cdot 10^{-5}$ M $K_3Fe(CN)_6$, $5 \cdot 10^{-4}$ M Na_2EDTA

Table S15. Rate Constants for the Reaction of Ascorbate with Hexacyanoferrate(III) Ion in Water at Various Concentrations of Tetramethylammonium Chloride (TMACl), at 25.0°C.

c(TMACl)/M	*I*	k_{HAsc^-}/M^{-1}s^{-1}
0.0005	0.0025	71.4 (0.4)
0.001	0.0030	75.2 (0.6)
0.003	0.0050	93.4 (0.3)
0.005	0.0070	106.2 (1.1)
0.007	0.0090	115.8 (0.9)
0.01	0.0120	129.6 (1.3)

$3 \cdot 10^{-4}$ M Na-ascorbate, $4 \cdot 10^{-5}$ M $K_3Fe(CN)_6$, $5 \cdot 10^{-4}$ M Na_2EDTA;

Table S16. Rate Constants for the Reaction of Ascorbate with Hexacyanoferrate(III) Ion in Water at Various Concentrations of Tetraethylammonium Chloride (TEACl), at 25.0°C.

c(TEACl)/M	I	k_{HAsc^-}/$M^{-1}s^{-1}$
0.0005	0.0025	73.3 (0.5)
0.001	0.0030	75.6 (0.7)
0.003	0.0050	84.1 (1.0)
0.005	0.0070	91.6 (0.8)
0.007	0.0090	98.7 (1.2)
0.01	0.0120	108.9 (1.3)

$3 \cdot 10^{-4}$ M Na-ascorbate, $4 \cdot 10^{-5}$ M $K_3Fe(CN)_6$, $5 \cdot 10^{-4}$ M Na_2EDTA

---

* references 19 – 21 in manuscript

** references 16, 31, 34, 35 in manuscript
